# Inducing In Situ M_2_ Macrophage Polarization for Tendinopathy Therapy through Microneedle Patch-Mediated Instant/Sustained Delivery of Rosmarinic Acid

**DOI:** 10.34133/bmr.0264

**Published:** 2025-11-08

**Authors:** Zheng Wang, Ying Chu, Yu Hu, Xue Fang, Jingyi Du, Mingshuang Li, Yuxin Zha, Jiabing Ran, Aixi Yu

**Affiliations:** ^1^ Department of Orthopedics Trauma and Microsurgery, Zhongnan Hospital of Wuhan University, Wuhan 430000, China.; ^2^ Hubei Clinical Medical Research Center of Trauma and Microsurgery, Wuhan 430000, China.; ^3^ Hubei Key Laboratory of Natural Products Research and Development, China Three Gorges University, Yichang 443002, China.; ^4^ College of Biological and Pharmaceutical Sciences, China Three Gorges University, Yichang 443002, China.

## Abstract

Tendinopathy markedly impairs patients’ quality of life, yet effective therapies remain limited. Conventional therapeutic strategies have largely overlooked the pivotal role of macrophages (M_φ_) and M_φ_-mediated immunoregulation in this condition. To address this, we designed a microneedle patch (MP) for the in situ delivery of rosmarinic acid (RosA), aiming to modulate local M_φ_ phenotype. We evaluated the therapeutic efficacy of this RosA-MP against tendinopathy and investigated the underlying mechanism. Briefly, the RosA-MP was fabricated by incorporating RosA into a microneedle array composed of poly(hydroxyethyl methacrylate-co-3-acrylamidophenyl boronic acid-co-(3-methacrylamidopropyl)trimethylammonium chloride) [poly(HEMA-co-3APBA-co-MAPTAC)], which was covalently linked to a flexible filter paper matrix. Formation of phenylboronic acid ester bonds between the 3APBA moieties and RosA endowed the RosA-MP with enhanced stiffness and skin penetration capability, facilitating sustained RosA release. Furthermore, the MAPTAC moieties provided instant hydrophilic swelling upon skin insertion, accelerating RosA delivery via increased internal osmotic pressure. In vitro and in vivo assays demonstrated that the RosA-MP simultaneously induced in situ M_2_ M_φ_ polarization and scavenged reactive oxygen species. Mechanistic investigation revealed that suppression of the NLRP3 inflammasome contributed to the rationale behind M_φ_ polarization. Using a collagenase type I-induced rat tendinopathy model, we assessed the therapeutic effects of the RosA-MP. Results confirmed that the RosA-MP effectively treated tendinopathy and exhibited significant advantages over injection therapy.

## Introduction

Tendinopathy, a prevalent tendon disorder with high morbidity rates, manifests histologically through abnormal cellular infiltration, dysregulated extracellular matrix remodeling, and disorganized collagen fiber alignment. At the molecular level, this condition is further characterized by increased microvascularization and enhanced sensory nerve innervation. These pathological changes collectively lead to significant deterioration in tendon biomechanical properties, clinically presenting as chronic pain, localized swelling, and functional impairment [1]. Tendinopathy frequently affects both upper and lower extremities, imposing substantial burdens on patients’ quality of life, particularly among athletic populations. While multiple etiological factors (e.g., mechanical overuse, pharmacological side effects, underlying metabolic disorders, and genetic predispositions) have been identified, the precise pathogenic mechanisms remain incompletely elucidated [2]. Traditional dogma held that inflammation played a negligible role in the progress of tendinopathy since there was no significant clinical evidence of a classic inflammatory event in tendinopathy (e.g., redness, tenderness, skin changes, or increased systemic inflammatory markers in blood) [3]. The results of microscopy also supported this assertion [2]. However, an increasing number of researchers have been gradually taking the opposite perspective in recent years because lots of inflammatory components have been found at acute tendon injury sites [3,4]. For instance, Legerlotz et al. [5] found that cyclooxygenase-2 (COX-2) and interleukin-6 (IL-6) expression was increased in ruptured Achilles tendon. Wu et al. [6] found that reducing inflammatory stimulus benefited for therapeutic outcomes in Achilles tendon rupture. Ward et al. [7] claimed that the fat pads adjacent to tendon and interfascicular matrix might be potential sources of key cytokines and inflammatory cells.

Since inflammatory reactions are not transient processes, establishing a sustained anti-inflammatory microenvironment appears essential for effective tendinopathy treatment [8]. The clinical strategies for inflammation management primarily involves 2 approaches: (a) oral anti-inflammatory drugs [9] and (b) closed therapy. However, both methods present significant limitations. The efficacy of oral anti-inflammatory drugs is often constrained by poor bioavailability and systemic side effects [10], while closed therapy may lead to structural complications such as tendon deformation, degeneration, and tissue adhesion, ultimately increasing the risk of tendon rupture [3,11]. Furthermore, the aforementioned therapies merely alleviate symptoms without halting progression or facilitating tendon regeneration [12]. Consequently, devising alternative therapeutic approaches to mitigate inflammation in tendinopathy is critically important and has garnered significant attention.

The severity of inflammation is strongly correlated with the extent of inflammatory cell infiltration; neutrophils and M_φ_ are representative cells infiltrated sequentially following tendon injury [12]. Neutrophil counts peak immediately post-injury, persist for approximately 7 days, and decline markedly after day 3 [4,13]. In contrast, M_φ_ concentrations remain elevated for extended periods, with increased levels observed up to 14 to 28 days post-injury [4]. Wong et al. documented distinct infiltration peaks: neutrophils at 1 to 5 days and M_φ_ at 21 days post-injury [14,15]. M_φ_ are highly plastic and could be polarized into 2 heterogeneous phenotypes: the “classically activated” M_1_ type and the “alternatively activated” M_2_ type [16,17]. Frenette et al. claimed that M_1_ M_φ_ were the culprit for the functional impairment in Achilles tendons [13,18]. Rodeo et al. proposed that M_1_ M_φ_ exacerbated inflammation and promoted reactive oxygen species (ROS) generation, aggravating tendon damage and inducing ectopic calcification, while M_2_ M_φ_ relieved inflammation and accelerated tendon healing [4,19,20]. Thus, persistently inducing M_1_-to-M_2_ transition of in situ M_φ_ has been considered a rational strategy for tendinopathy treatment, which takes effect through simultaneously reducing inflammatory reaction/ROS accumulation and promoting tendon healing.

To date, several strategies have been reported to induce M_1_-to-M_2_ transition of in situ M_φ_ for tendinopathy treatment, yet their clinical applicability remains limited. In situ implantation of immunoregulatory biomaterials faces challenges of invasiveness. For example, the surgical deployment of polycaprolactone fiber scaffold with tailored topographical/mechanical cues [21], magnetic nanoparticle-incorporated polycaprolactone membranes [22], or poly(ɛ-caprolactone)/protocatechuic aldehyde hydrogel composite patches [23] requires specialized surgical expertise. Consequently, injectable immunomodulatory biomaterials have emerged as promising alternatives, such as small/large extracellular vesicles from human induced pluripotent stem cell (iPSC)-derived MSCs [24], lipid-polymer nanoparticles co-loaded with budesonide and serpine1 siRNA [25], parishin A-loaded mesoporous silica nanoparticles [26], injectable decellularized tendon-derived extracellular matrix hydrogels [27], and 3-dimensional (3D)-printed hydrogel particles laden with platelet-rich plasma and tendon-derived stem cells [28]. However, in situ injection of these materials still requires professional surgical administration. Microneedle patch (MP), a transdermal drug delivery platform characterized by noninvasiveness, painlessness, simple operation, controllable drug release, and versatile payload capacity, represents a promising therapeutic strategy for tendinopathy [29–32]. Nevertheless, current MP-based approaches for tendinopathy primarily target tendon cells and progenitor cells, while the pivotal immunomodulatory roles of M_φ_ and M_φ_-mediated immune regulation in therapy remain largely underestimated.

**Fig. 1.**
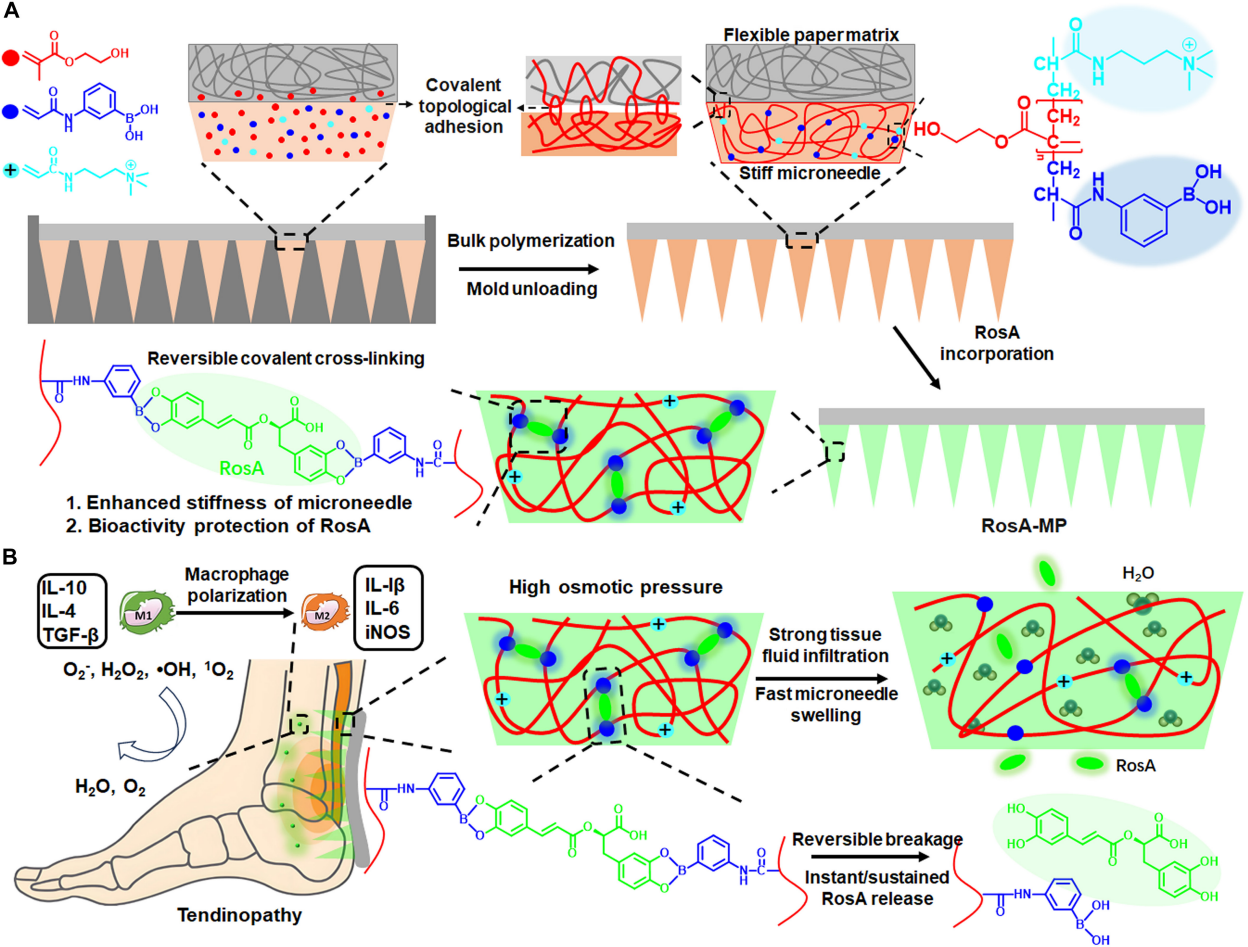
(A) Schematic diagram of the RosA-MP structural composition. (B) Therapeutic application of the RosA-MP for tendinopathy and its underlying mechanism.

Accordingly, we developed an MP-based transdermal drug delivery system to foster an immunoregulatory microenvironment that sustains M_2_ M_φ_ polarization at the tendinopathy site, subsequently assessing its therapeutic efficacy and investigating the underlying mechanisms. In this scheme (Fig. 1), the MP was designed based on our previous work: (a) the pristine poly(hydroxyethyl methacrylate-co-3-acrylamidophenyl boronic acid-co-(3-methacrylamidopropyl)trimethylammonium chloride) [poly(HEMA-co-3APBA-co-MAPTAC)] microneedle array was synthesized via bulk co-polymerization of HEMA, 3APBA, and MAPTAC [16]. Subsequently, this array was immobilized onto a filter paper matrix through monomer permeation-mediated covalent topological adhesion to construct the MP [33]; (b) the resulting poly(HEMA-co-3APBA-co-MAPTAC) MP (designated B-MP) underwent equilibrium swelling in a rosmarinic acid (RosA)-containing ethanol solution, followed by ethanol evaporation, yielding the final RosA-MP [17]. The filter paper matrix confers high flexibility and foldability to the RosA-MP, enabling adaptation to the 3D geometry of tendons. RosA, a natural phenolic compound characterized by dual catechol groups, exhibits potent anti-inflammatory activity validated across multiple inflammatory conditions including arthritis, colitis, atopic dermatitis, asthma, allergic rhinitis, periodontal disease, acute pancreatitis, mastitis, paw edema, and seasonal allergic rhinoconjunctivitis [34]. Unlike conventional anti-inflammatory agents, RosA uniquely achieves ‌dual modulation: (a) direct reduction of neutrophil and eosinophil infiltration at inflammatory sites, and (b) efficient induction of in situ M_2_ M_φ_ polarization [35,36]. Furthermore, RosA demonstrates complementary pharmacological properties encompassing antioxidant, antimicrobial, antidiabetic, and antiallergic activities, while maintaining a favorable safety profile with negligible side effects [37,38]. In this system, RosA molecules also serve dual roles: (a) crosslinking the poly(HEMA-co-3APBA-co-MAPTAC) chains to enhance microneedle stiffness and thereby skin penetration capability, and (b) undergoing slow therapeutic release via cleavage of reversible boronic ester bonds for tendinopathy treatment. Concurrently, MAPTAC moieties elevate internal osmotic pressure within the microneedles, accelerating hygroscopic swelling and subsequent RosA release.

In this study, we investigated the composition, structure, RosA loading efficiency, and mechanical strength of the microneedles (RosA-MP). The release kinetics of RosA molecules from the RosA-MP were determined by plotting cumulative release curves, with the underlying rationale investigated through curve fitting and analysis of the fitted parameters. In vitro M_φ_ coculture assays (e.g., immunofluorescence staining, flow cytometry, Western blotting, and 2′7′-dichlorodihydrofluorescein diacetate [DCFH-DA] fluorescence staining) were used to assess the RosA-MP’s induction of M_2_ Mφ polarization and ROS capture. Gene sequencing was applied to elucidate the mechanism by which the RosA-MP induces M_2_ M_φ_ polarization. Achilles tendinitis model was established in rats via in situ injecting collagenase I (Col1) solution. Following the RosA-MP treatment, its in vivo immunoregulatory function, antioxidant capacity, and therapeutic efficacy against tendinitis were comprehensively evaluated using hematoxylin and eosin (H&E) staining, Masson’s trichrome staining, and immunohistochemical analysis*.* This study reports a novel MP with significant therapeutic potential for clinical management of tendinopathy.

## Materials and Methods

### Materials

HEMA (99%) was purchased from Aladdin Biochemical Technology Co., Ltd. (Shanghai, China). *N*,*N*,*N*′,*N*′-tetramethylethylenediamine (TEMED, ≥ 98%) was purchased from Kemiou Chemical Reagent Co., Ltd. (Tianjin, China). RosA (99%), 3APBA (99%), *N*,*N*′-methylene diacrylamide (MBAA, 99%), benzoin dimethyl ether (DMPA, 99%), and MAPTAC (99%) were bought from Shanghai Macklin Biochemical Co., Ltd. (Shanghai, China). Ethanol was received from Sinopharm Chemical Reagent Co., Ltd. (Shanghai, China). All reagents were used as received without further purification. Deionized water was used throughout the experiment.

### Fabrication of the RosA-MP

3APBA, DMPA, MAPTAC, MBAA, and TEMED were added to a certain volume of HEMA solution and then subjected to ultrasonication until all the solids were dissolved (3 min, 25 °C). The initial feeding composition for HEMA, 3APBA, MAPTAC, MBAA, DMPA, and TEMED can be found in Table S1. Next, the as-prepared solution (around 1.004 ml) was slowly dripped into a polydimethylsiloxane (PDMS) mold (25 °C). Figure S1 shows the 3-view drawing of the PDMS. The depth of conical pits of the PDMS mold was 1,000 μm while the height of the mixed solution was maintained at around 1,100 μm (Fig. S1). Then, the whole mold was placed in a vacuum oven (−0.09 to 0.1 MPa) and maintained for 30 min at 25 °C. Next, a filter paper (thickness: 340 μm) was firstly wetted with ethanol and then covered on the solution (Fig. S1) and the whole mold was placed in the vacuum oven (−0.09 to 0.1 MPa) for 30 min again at 25 °C. Afterwards, the solution covered with a filter paper was exposed to ultraviolet light (254 nm, 120,000 μJ/cm) for 1 h (25 °C), during which radical copolymerization happened. Here, MBAA, DMPA, and TEMED served as a crosslinker, initiator, and accelerator for radical polymerization, respectively. After mold unloading, the as-prepared B-MP was then soaked into water/ethanol (v/v = 1:1) solution for 24 h to remove DMPA, TEMED, unreacted monomers, and other impurities. Subsequently, the swollen B-MP was placed in an oven (37 °C) to completely remove both moisture and ethanol. The resultant dehydrated/dealcoholized B-MP was then soaked in an ethanol solution of RosA. Herein, the concentration of the ethanol solution of RosA was kept at 0.41 mg/ml, which was determined through investing the equilibrium swelling ratio of the B-MP in phosphate-buffered saline (PBS; Table S2) and ethanol (Table S3). Until swelling equilibrium was reached, the resultant RosA-MP was rinsed 3 times with water to remove the remaining RosA on the surface of microneedles. The as-received sample was dealcoholized in a vacuum oven (−0.09 to 0.1 MPa, 25 °C, 2 h). Finally, the RosA-MP was obtained. Poly(HEMA)/RosA MP and poly(HEMA-co-3APBA)/RosA MP were also fabricated according to similar procedures and utilized as controls in this work.

### Characterizations

#### Fourier transform infrared spectrum

Fourier transform infrared spectrum (FTIR) (Nicolet 550 II, Thermo Fisher Scientific, USA) was employed to analyze the composition of the RosA-MP and investigate interactions between functional groups. The RosA-MP was first ground into powder and then subjected to FTIR analysis using a KBr pellet method. Additionally, FTIR spectra of RosA and B-MP were acquired.

#### X-ray diffraction

X-ray diffraction (XRD) analysis (Panalytical BV, Netherlands) was performed to characterize the crystalline phases of RosA-MP. The RosA-MP was directly subjected to XRD measurement, with the B-MP and RosA also analyzed for comparison. The instrument operated under CuK_0_ radiation at 40 kV and 40 mA using a rotating anode generator. Data acquisition employed a 0.1° step size over a 2θ range of 5° to 90°.

#### X-ray photoelectron spectrometer

The formation of phenylboronic acid ester bonds between the 3APBA moieties and RosA molecules in the RosA-MP was verified using x-ray photoelectron spectrometer (XPS) analysis (Escalab 250 Xi, Thermo Fisher Scientific). The verification process involved recording survey spectra of both the B-MP and the RosA-MP, with the C1s peak at 285 eV serving as the binding energy calibration reference. For detailed characterization, the high-resolution O1s spectrum was deconvoluted into subcomponents using Avantage software.

#### Thermogravimetric analysis and differential scanning calorimetry

Thermogravimetric (TG) analysis (NETZSCH TG/DSC, Germany) was employed to quantify the RosA content in the RosA-MP, while differential scanning calorimetry (DSC) was used to evaluate the protective effect of phenylboronic acid ester bonds on RosA’s catechol structure. The experimental procedure involved (a) grinding the RosA-MP into powder prior to TG testing, and (b) parallel testing of the B-MP and pure RosA as controls. All specimens were heated from 25 to 800 °C at a constant rate of 10 °C/min under ambient air conditions.

#### Scanning electron microscope (SEM)

The surface morphology of the RosA-MP was analyzed using a scanning electron microscope (SEM; Sigma, Zeiss, Germany). Prior to imaging, the sample was sputter-coated with gold for 120 s. SEM observation was then conducted at an accelerating voltage of 5 kV and a working distance of 7.5 mm.

#### Investigation of the mechanical properties

Compression tests and depth-sensing indentation (DSI) were used to evaluate the skin penetration capability of the microneedles. For compression tests, the RosA-MPs were compressed using a Universal Testing Machine (Qinji QJ210A, Shanghai, China), with loading directly on the microneedles at a crosshead speed of 1 mm/min. The force–displacement curves were recorded directly by the machine, enabling determination of the fracture force (*F*_f_) and fracture displacement (*D*_f_). The fracture energy (*W*_f_) was derived from the area under the force–displacement curve of a sample. The fracture force per microneedle *F*_f,m_ was calculated by dividing the Ff of the entire MP array by 100. Similarly, the fracture energy of per microneedle *W*_f,m_ was obtained by dividing the *W*_f_ of the MP array by 100.

$$W_{f}=\int_{0}^{D_{f}}\mathrm{Fd}D$$ (1)

$$F_{f,m}=\frac{F_{f}}{100}$$ (2)

$$W_{f,m}=\frac{W_{f}}{100}$$ (3)

where *F* and *D* denote force and displacement, respectively.

The DSI tests were conducted using a Nano Indenter G200 (Agilent, USA) with a diamond indenter of regular geometry featuring a tip radius below 20 nm. The indenter was carefully positioned on the surface of either bulk poly(HEMA-co-3APBA-MATPAC) or poly(HEMA-co-3APBA-MAPTAC)/RosA specimens, followed by the application of gradually increased external force. After reaching the maximum load of 10 mN, the force was systematically unloaded, during which the load–displacement curve was recorded. Prior to testing, both the indenter area function and the load frame compliance were calibrated using a fused silica standard sample in compliance with ISO-14577-1:2015 specifications.

### Investigation of the releasing behaviors of RosA from the RosA-MP

The RosA-MP was placed in a dialysis bag (molecular weight cutoff: 3,500 Da) and immersed in 10 ml of PBS (37 °C) under gentle agitation (120 rpm). At predetermined intervals, 10 ml of PBS was collected and replaced with 10 ml of fresh PBS. The collected PBS was analyzed by high-performance liquid chromatography (HPLC) (Thermo Field SRD-3600, USA), and the released RosA concentration was quantified using a standard curve method (Fig. S2). Results, expressed as cumulative release curves, represent the average of triplicate measurements. Seven mathematical models were applied to fit the RosA release curves. To investigate the release mechanism, the RosA-MP release curves were compared at different pH values (pH 5.5, 7.4, and 9.3), along with those of the PHEMA/RosA and the poly(HEMA-co-3APBA)/RosA. For HPLC tests, octadecylsilane bonded silica gel was used as filler. The mobile phase was a mixture of methanol and 0.1% trifluoroacetic acid (40:60). Detection wavelength was maintained at 330 nm and flow rate was kept at 1.0 ml·min^−1^.

### In vitro swelling assay

The swelling ratio–time curve of the RosA-MP was plotted using a gravimetric method, beginning with weighing the initial mass (*m*_0_), followed by immersion in PBS at 37 °C and pH 7.4; at each time point *t*, the mass (*m_t_*) was reweighed to determine the swelling ratio based on mass changes_._ The swelling ratio of the RosA-MP was calculated using the following equation:

$$\text{Swelling ratio}=\frac{m_{t}-m_{0}}{m_{0}}$$ (4)

The swelling ratio–time curves of the PHEMA/RosA and the poly(HEMA-co-3APBA)/RosA were plotted following identical gravimetric procedures. Additionally, the equilibrium swelling ratios of the poly(HEMA-co-3APBA-MAPTAC) and the poly(HEMA-co-3PABA) were also investigated.

### Cell culture and treatment

The murine macrophage cell line Raw 264.7 (Procell, Wuhan, China) was cultured in high-glucose Dulbecco’s Modified Eagle Medium (DMEM) (Procell, China) supplemented with 10% fetal bovine serum (FBS; Procell, China) under standard conditions (37 °C, 5% CO_2_). For the coculture assay, the MPs were loaded into a 0.22-μm pore-sized transwell chamber (Costar, USA) at a concentration of 20 mg/ml, while Raw 264.7 cells were maintained in the lower compartment to establish a noncontact coculture system. Primary bone marrow-derived macrophages (BMDMs) were isolated from femurs and tibias of 6- to 8-week-old C57BL/6 mice. ‌Bone marrow cells were extracted by flushing with sterile PBS, filtered through a 70-μm cell strainer, and centrifuged at 300 × *g* for 5 min.‌ The cells were then cultured in high-glucose DMEM supplemented with 10% FBS and 20 ng/ml recombinant mouse macrophage colony-stimulating factor (M-CSF; Peprotech, USA) for 7 days. ‌During differentiation, the medium was replaced every 2 to 3 days to remove nonadherent cells and replenish growth factors.‌ Mature BMDMs were harvested using the same protocol as applied to Raw 264.7 cells for subsequent experiments.

#### Biocompatibility and hemocompatibility tests of the RosA-MP

Cell viability was quantitatively evaluated using a CCK-8 kit (Biosharp, China) to determine the biocompatibility of the RosA-MP. Briefly, Raw 264.7 cells were seeded in 6-well plates at a density of 1 × 10^5^ cells/ml and exposed to either the B-MP or the RosA-MP, with a parallel negative control group containing only complete culture medium. Following 24-, 48-, and 72-h incubation periods, the original medium was replaced with 2 ml of fresh medium containing 10% (v/v) CCK-8 solution (200 μl of reagent in 2 ml of medium). After an additional 2-h incubation at 37 °C, 100-μl aliquots of supernatant were transferred to 96-well plates for absorbance measurement at 450 nm using a Thermo Fisher Scientific microplate reader.

The hemolytic potential of the RosA-MP was systematically assessed according to ISO 10993-4 standards using a rabbit erythrocyte model. Fresh whole blood from healthy New Zealand white rabbits was collected in heparinized tubes and centrifuged at 1,500 rpm for 10 min to isolate red blood cells (RBCs). After 3 washes with PBS (pH 7.4), the RBCs were resuspended to achieve a 2% (v/v) hematocrit. Test groups consisted of RosA-MP leaching solutions at 5 concentrations (10, 20, 30, 50, and 100 mg/ml), with normal saline and 0.1% Triton X-100 serving as negative and positive controls, respectively. Each experimental group (200 μl of RBC suspension + 800 μl of test solution) was incubated at 37°C for 30 min with gentle agitation. Following centrifugation at 3,000 rpm for 5 min, supernatant absorbance at 570 nm was measured using a Thermo Fisher Scientific Varioskan LUX microplate reader.

The hemolysis rate (%) was calculated as:

$$\text{Hemolysis}=\frac{{\mathrm{OD}}_{\text{sample}}-{\mathrm{OD}}_{\text{negative}}}{{\mathrm{OD}}_{\text{positive}}-{\mathrm{OD}}_{\text{negative}}}\times 100\%$$ (5)

where values >5% were considered clinically significant according to ASTM F756-17.

#### Investigation of the effect of the RosA-MP on inducing M_φ_ polarization

To systematically investigate the immunomodulatory effects of the RosA-MP on M_φ_ polarization, Raw 264.7 cells were seeded in 6-well plates (1 × 10^5^ cells/ml) and exposed to 4 experimental conditions for 72 h in a humidified 5% CO_2_ incubator at 37 °C: (a) complete DMEM medium (Control), (b) medium containing 100 ng/ml ultrapure lipopolysaccharide (LPS) as the inflammatory stimulus [36], (c) LPS medium supplemented with blank MP extracts (B-MP), and (d) LPS medium with the RosA-MP extracts. Post-incubation, cells were processed for multimodal analysis: (a) immunofluorescence staining using anti-CD86, anti-iNOS, anti-Arg1, and anti-CD206 antibodies with DAPI counterstaining; (b) Western blotting with RIPA lysates separated on 10% SDS-PAGE gels; and (c) flow cytometry analyzing 10,000 events per sample. Concurrently, culture supernatants were assessed for cytokine secretion using enzyme-linked immunosorbent assay (ELISA) kits (Meimian, China) with absorbance measured at 450/570 nm dual wavelengths, following the manufacturer’s standardized protocol including spike-and-recovery validation. To validate the translational potential of the RosA-MP, its effects on M_φ_ polarization were systematically assessed in primary mouse BMDMs ‌using identical experimental protocols to those applied in Raw 264.7 cells‌. ‌This parallel evaluation‌ not only confirmed the robustness of the initial findings ‌but also enhanced their clinical relevance‌, given that primary BMDMs better recapitulate human M_φ_ physiology ‌compared to immortalized cell lines‌.

##### Immunofluorescence staining

Cells were incubated overnight at 4 °C with primary antibodies against CD86 (M_1_) and CD206 (M_2_) for Raw 264.7 cells, and against iNOS (M_1_) and Arg1 (M_2_) for BMDMs. Following 3 PBS washes to remove unbound antibodies, the cells were subsequently incubated for 1 h at room temperature with species-matched secondary antibodies (antibody specifications provided in Table S4). Nuclear counterstaining was performed using DAPI (4′,6-diamidino-2-phenylindole) before fluorescence imaging with a fluorescence microscope (Olympus, Japan). Quantitative analysis of fluorescence intensity for both markers was conducted using the ImageJ software (NIH) with consistent threshold settings across all samples to ensure comparability.

##### RNA sequencing and bioinformatic data analysis

To elucidate the underlying mechanisms of M_φ_ polarization, total RNA was extracted from Raw 264.7 cells (*n* = 3) after 3 days of coculture with the RosA-MP extracts using the Qiagen RNeasy Mini Kit (Qiagen, Hilden, Germany). Raw 264.7 cells treated with LPS (10 ng/ml) was used as control. After RNA extraction, RNA sequencing was conducted using the Illumina sequencing platform. Differentially expressed genes (DEGs) were identified through DESeq2 v1.6.3, applying a threshold of *q* ≤ 0.05 and |log2_ratio| ≥ 1. Functional predictions for DEGs were performed using Gene Ontology (GO) and Kyoto Encyclopedia of Genes and Genomes (KEGG) enrichment analyses.

##### qPCR analysis

Total RNA was isolated from treated macrophages using TRIzol reagent (Invitrogen, USA) following the manufacturer’s protocol. ‌RNA concentration and purity were determined by measuring absorbance at 260/280 nm using a NanoDrop spectrophotometer.‌ Complementary DNA (cDNA) was synthesized from 1 μg of total RNA with a PrimeScript RT reagent kit (Takara, Japan). Quantitative real-time PCR was performed in triplicate using SYBR Green Master Mix (Applied Biosystems, USA) on a StepOnePlus system (Applied Biosystems, USA), ‌with cycling conditions as follows: 95 °C for 10 min, followed by 40 cycles of 95 °C for 15 s and 60 °C for 1 min.‌ Gene expression of M_φ_ polarization markers (iNOS and TNF-α for the M_1_ phenotype; Arg1 and TGF-β for the M_2_ phenotype) and the endogenous control GAPDH was analyzed using ‌prevalidated primers‌. Relative quantification was calculated via the 2^–ΔΔCt^ method ‌with inter-run calibrators to ensure reproducibility.

##### Western blotting

Protein samples extracted from Raw 264.7 cells using RIPA lysis buffer (Solarbio, China) were quantified by bicinchoninic acid (BCA) assay before electrophoresis. Equal amounts of protein (30 μg per lane) were separated on 10% SDS-polyacrylamide gels and subsequently transferred to PVDF membranes (0.45 μm pore size; Millipore, USA) using a semi-dry transfer system. After blocking with 5% nonfat milk in TBST (Tris-buffered saline with 0.1% Tween-20) for 1 h at room temperature, the membranes were incubated overnight at 4°C with the following primary antibodies: anti-CD86, anti-CD206, anti-NLRP3, anti-Pro-caspase-1, anti-Cleaved-caspase-1, and anti-IL-1β (detailed antibody information in Table S5). Following 3 10-min TBST washes, membranes were incubated with horseradish peroxidase (HRP)-conjugated secondary antibodies for 1 h at room temperature. Protein bands were visualized using an Enhanced Chemiluminescence Detection Kit (Baiqiandu, China) and quantified by ImageJ software (NIH) with β-actin serving as the loading control.

#### Animal model establishment

All animal experiments were conducted in strict compliance with the NIH Guide for the Care and Use of Laboratory Animals and approved by the Experimental Animal Welfare Ethics Committee of Zhongnan Hospital, Wuhan University (Ethical Approval No. ZN2023197). A total of 50 male Sprague–Dawley rats (8 weeks old, 250 ± 20 g) were randomly allocated into 5 experimental groups (*n* = 10 per group): (a) negative control (untreated), (b) tendinopathy model, (c) the B-MP treatment, (d) RosA injection (100 μg), and (e) the RosA-MP treatment. As to the RosA-Injection group, the rats received 100 μg of RosA by injection once per week for 4 consecutive weeks. Following a 7-day acclimatization period with ad libitum access to food and water, tendinopathy was induced in all groups except controls by ultrasound-guided injection of 60 μl of collagenase I solution (5 mg/ml in PBS) into the left Achilles tendon [9,39]. Therapeutic interventions commenced 7 days post-modeling, with the B-MP and RosA-MP groups receiving respective biomaterial treatments, while the RosA-Injection group received direct drug administration. After 4 weeks of treatment, animals were euthanized by CO_2_ asphyxiation for tendon harvest. Comprehensive evaluation included (a) macroscopic assessment of tendon morphology, (b) histological analyses, (c) immunohistochemical detection of collagen types, and (d) biomechanical testing.

##### Histological, immunohistochemical, and immunofluorescent staining

Tendon specimens were fixed in 4% paraformaldehyde for 24 h at 4°C, followed by gradient ethanol dehydration (70% to 100%), xylene clearing, and paraffin embedding. Serial sections (5 μm thickness) were prepared using a rotary microtome (Leica RM2235) for histological assessments. For general morphology evaluation, sections were subjected to standard H&E staining, while collagen fiber organization was analyzed using Masson’s trichrome staining. Picrosirius red staining was performed with 0.1% Sirius red in saturated picric acid solution (1 h incubation). The alignment of collagen fibers in tendon microstructure was quantitatively evaluated by analyzing histological images acquired under polarized light microscopy using ImageJ. The Orientation Index was calculated, where a value of 1 represents perfectly aligned fibers and 0 indicates random orientation.

Immunohistochemical analysis involved deparaffinization, antigen retrieval (10 mM citrate buffer, pH 6.0, 95°C for 20 min), and blocking with 3% bovine serum albumin. Primary antibodies against Col1 and Col3 were applied overnight at 4 °C, followed by HRP-conjugated secondary antibody incubation (1 h, RT). Diaminobenzidine was used as chromogen, with hematoxylin counterstaining. Quantitative analysis was performed using ImageJ software (NIH) by measuring integrated optical density in 5 randomly selected fields per section.

For oxidative stress evaluation, cryosections (7 μM) were incubated with 2.5 μM dihydroethidium (DHE) for 30 min at 37°C in dark. M_φ_ polarization was assessed by immunofluorescence double staining using anti-CD86 together with anti-F4/80 to identify M_1_-like M_φ_, and anti-CD206 together with anti-F4/80 to identify M_2_-like M_φ_, with Alexa Fluor-conjugated secondary antibodies. Nuclei were counterstained with DAPI, and fluorescence images were acquired using a confocal microscope (Olympus) with consistent exposure settings across samples. All antibodies and reagents are detailed in Table S6.

##### Biomechanical testing

For biomechanical characterization, harvested tendon specimens were securely mounted on a custom-designed testing fixture using dual cryo-clamps (maintained at −20 °C) to prevent slippage during mechanical loading. Mechanical properties were evaluated using a servo-hydraulic testing system (Bose ElectroForce 3220-AT, USA) equipped with a 50-N load cell (accuracy ±0.1%). Specimens were subjected to uniaxial tensile testing at a constant displacement rate of 100% strain per second (corresponding to 5 mm/min for average tendon length) until complete rupture occurred. The peak force at failure was automatically recorded by the system’s WinTest software (v7.1), with simultaneous acquisition of displacement data at 1,000 Hz sampling frequency. Stress–strain curves were generated by normalizing force values to the tendon’s cross-sectional area (measured via digital caliper prior to testing) and strain was calculated as the ratio of displacement to original gauge length (20 mm between clamps).

#### Statistical analysis

All relevant data were expressed as mean ± standard deviation (SD) and analyzed using GraphPad Prism 9. Group comparisons were conducted through one-way analysis of variance followed by Tukey’s post hoc test. A *P* value < 0.05 was considered statistically significant (^*^P < 0.05; ^**^P < 0.01; ^***^P < 0.005; ^****^P < 0.001).

## Results and Discussion

### Morphology, composition, and structure of the RosA-MP

**Fig. 2.**
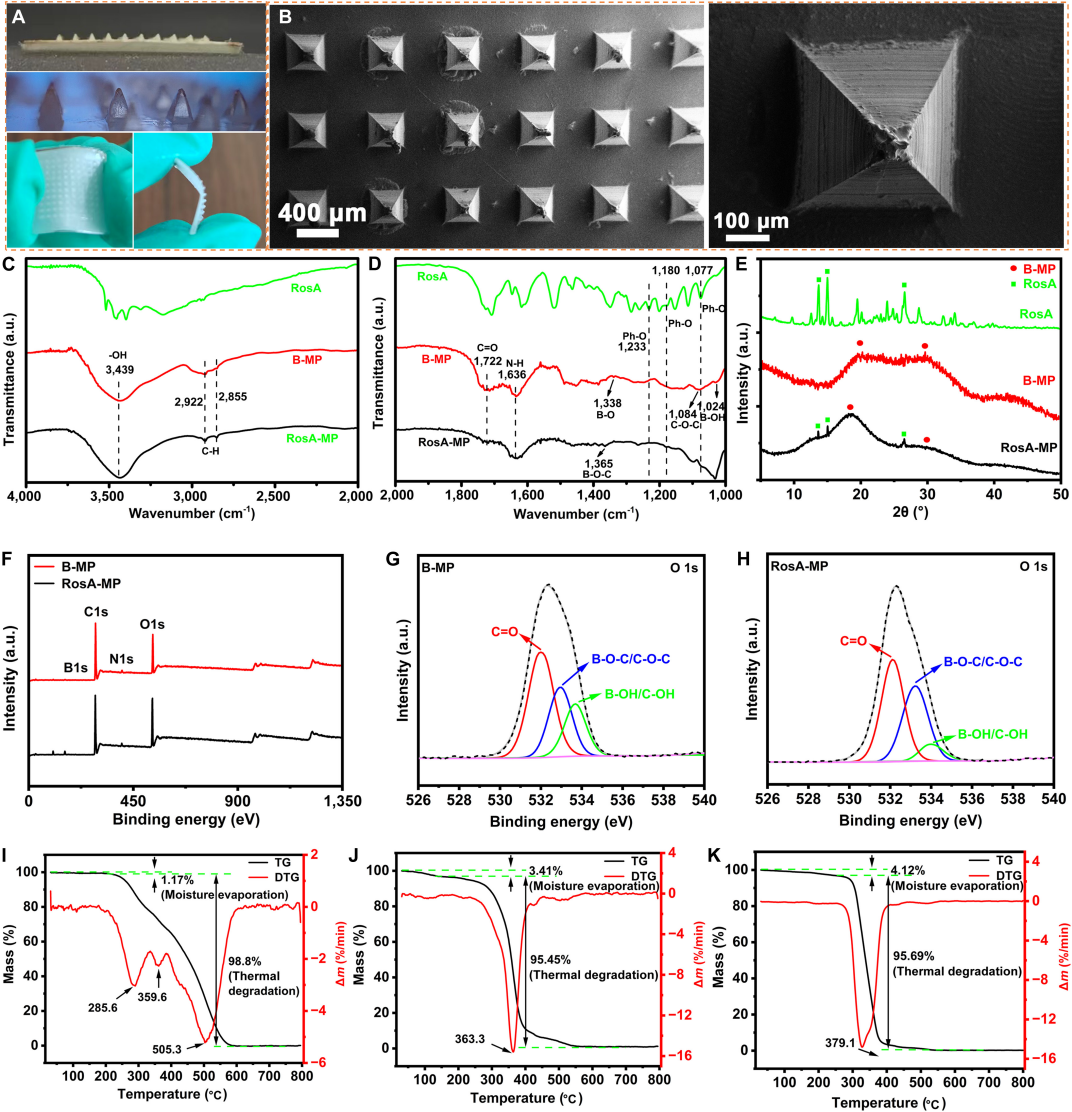
(A) Digital photographs and (B) SEM images of the RosA-MP. FTIR spectra of RosA, the B-MP, and the Rosa-MP in wavenumber ranges of (C) 4,000 to 2,000 cm^−1^ and (D) 2,000 to 1,000 cm^−1^. (E) XRD spectra of RosA, the B-MP, and the RosA-MP. (F) XPS spectra of the B-MP and the RosA-MP. (G and H) O1s spectra of the B-MP and the RosA-MP, respectively. TG-DTG curves of (I) RosA, (J) the B-MP, and (K) the RosA-MP.

Figure 2A and B show the digital photographs and SEM images of the as-prepared RosA-MP, respectively. It could be found that (a) the microneedles were securely bonded to the filter paper matrix and the RosA-MP could be freely bent and folded, demonstrating excellent 3D structural adaptivity; (b) the microneedles were orderly arrayed and had a rectangular pyramid shape. The tips of the microneedles were very sharp, beneficial for penetrating skin. Figure 2C and D demonstrate the FTIR spectra of RosA, the B-MP, and the RosA-MP. For the B-MP, the peaks at 3,439 and 1,084 cm^−1^ were attributed to the stretching of -OH and C-O-C of the HEMA moieties, respectively [40]; the bands at 1,722 and 1,636 cm^−1^ were attributed to the vibration of the amide I (C=O) and amide II (N-H) from the 3APBA and MAPTAC moieties [41]; the peaks at 1,338 and 1,024 cm^−1^ were ascribed to the B-O and B-O-H of the 3APBA moieties [42]; the bands at 2,922 and 2,855 cm^−1^ were assigned to the C-H of the alkylammonium cations of the MAPTAC moieties [43]. The above results proved that the co-polymerization of the HEMA, 3APBA, and MAPTAC formed the B-MP.

Regarding RosA, the absorbance peaks at 1,233, 1,180, and 1,077 cm^−1^ were owing to the C-O stretching vibration of phenolic hydroxyl groups. These peaks persisted in the FTIR spectrum of the RosA-MP, confirming RosA incorporation. The new peak at 1,365 cm^−1^ in the RosA-MP directly evidenced phenylboronic acid ester bond formation between the 3APBA moieties and RosA molecules [44]. The XRD spectra of RosA, the B-MP, and the RosA-MP also proved the incorporation of RosA molecules into the RosA-MP. In addition, no strong diffraction peaks of RosA were observed in the XRD spectrum of the RosA-MP, showing the homogeneous distribution of RosA molecules within the RosA-MP (Fig. 2E). To further verify the formation of phenylboronic acid ester bonds between the 3APBA moieties and RosA molecules in the RosA-MP, XPS analysis of the B-MP and the RosA-MP was applied (Fig. 2F), and the O1s spectra were separated and fitted in detail (Fig. 2G and H). It could be found that the C-O-B/C-O-C ratio was increased from 29.25 to 38.90 while the B-OH/C-OH ratio was decreased from 21.17 to 8.19 (Table 1), strongly proving the formation of phenylboronic acid ester bonds within the RosA-MP.

**Table 1.** Percentages of different atoms and functional groups of the B-MP and the RosA-MP obtained from the XPS spectra

Sample	B-MP	RosA-MP
Atomic composition		
O (%)	20.77	23.97
C (%)	79.01	75.81
B (%)	0.22	0.22
O-based functional group (%)		
C=O	49.59	52.19
C-O-B/C-O-C	29.25	38.90
C-OH/B-OH	21.17	8.91

To determine the RosA content within the RosA-MP, the TG curves of RosA (Fig. 2I), the B-MP (Fig. 2J), and the RosA-MP (Fig. 2K) were obtained and analyzed in detail. From the TG curve of RosA (Fig. 2I), the mass loss percentage of RosA in the temperature range of 20 to 800 °C was determined to be

$$W_{\text{loss}}\left(\text{RosA}\right)=\frac{100-1.17-0.03}{100}\times 100\%=98.8\%$$ (6)

From the TG curve of the B-MP (Fig. 2J), the mass loss percentage of the B-MP in the temperature range of 20 to 800 °C was determined to be

$$W_{\text{loss}}\left(B-\mathrm{MP}\right)=\frac{100-3.41-1.14}{100}\times 100\%=95.45\%$$ (7)

From the TG curve of the RosA-MP (Fig. 2K), the mass loss percentage of the RosA-MP in the temperature range of 20 to 800 °C was determined to be

$$W_{\text{loss}}\left(\text{RosA}-\mathrm{MP}\right)=\frac{100-4.12-0.19}{100}\times 100\%=95.69\%$$ (8)

It was assumed that 100 g of dehydrated RosA-MP contained *X* g RosA and *Y* g B-MP. In this regard, there are 3 equations:

X+Y=100 (9)

$$W_{\text{loss}}\left(\text{RosA}\right)\cdot X+W_{\text{loss}}\left(B-\mathrm{MP}\right)\cdot Y=95.69$$ (10)

Thus, the RosA content of the RosA-MP was determined to be

$$W_{\text{RosA}}=\frac{X}{100}\times 100\%=7.16\%$$ (11)

Furthermore, the DSC curves of RosA, the B-MP, and the RosA-MP were plotted at the same time (Fig. S3). It could be found that the first exothermic peak of RosA, which corresponded to the oxidization of catechol group into benzoquinone [36], disappeared in the DSC curve of the RosA-MP. It was inferred that the phenylboronic acid ester bonds prevented the RosA molecules from losing the bioactivity.

### Mechanical properties and RosA releasing behaviors of the RosA-MP

**Fig. 3.**
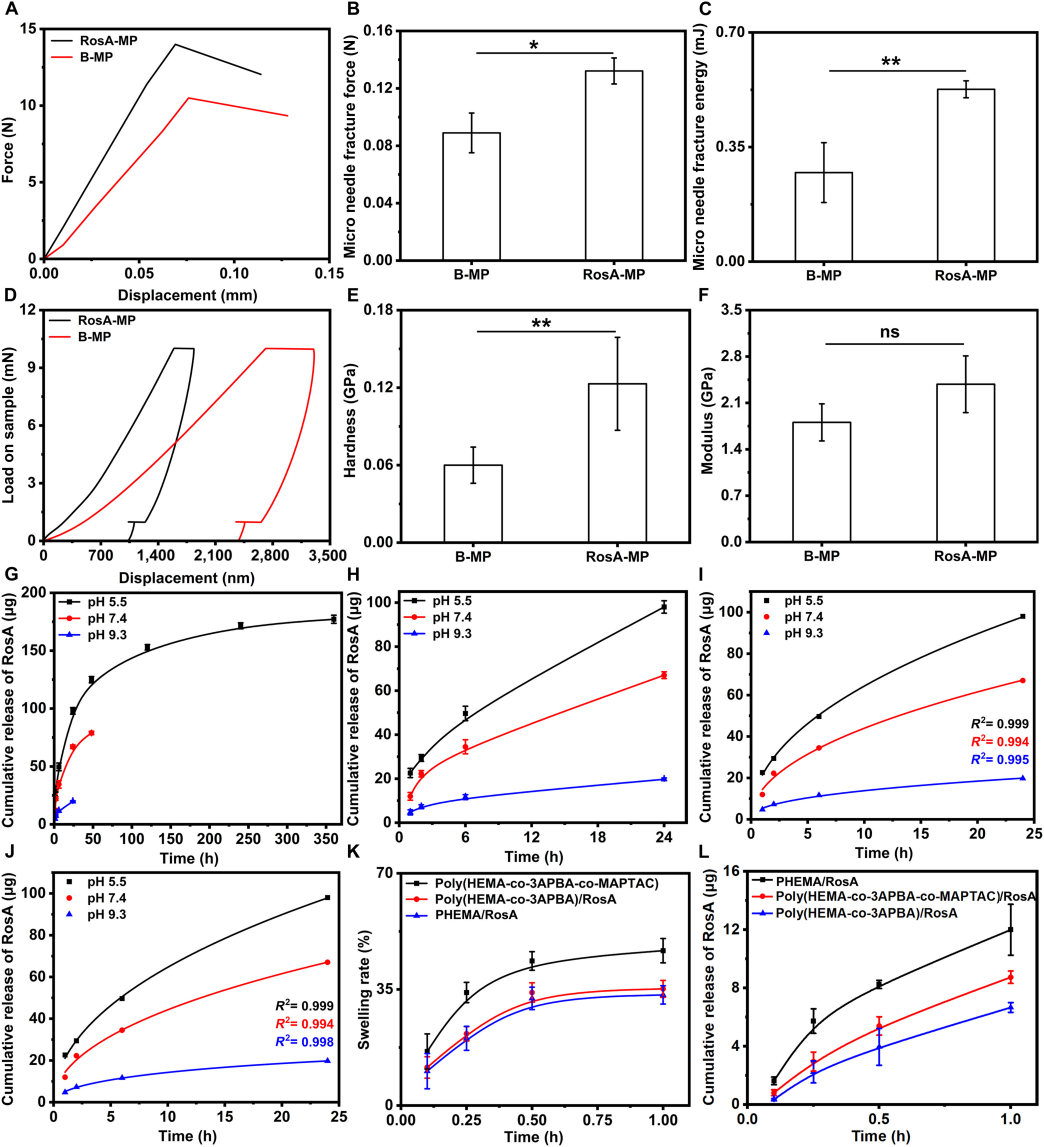
(A) Representative force–displacement curves of the B-MP and the RosA-MP uniaxial compression loading (load directly applied to microneedles). (B and C) Fracture force and fracture energy derived from (A). (D) Representative force–displacement curves of the B-MP and the RosA-MP in DSI assays [load directedly applied to the bulk poly(HEMA-co-3APBA-MATPAC) or poly(HEMA-co-3APBA-MAPTAC)/RosA specimens]. (E and F) Hardness and elastic modulus calculated from (D). (G and H) Cumulative RosA release profiles from the RosA-MP in PBS at varied pH values. (I and J) Fitted curves for (H) using Korsmeyer–Peppas and Kopcha models. (K) Swelling ratio–time profiles for the PHEMA, the poly(HEMA-co-3APBA), and the poly(HEMA-co-3APBA-co-MAPTAC). (L) Cumulative RosA release from the PHEMA/RosA MP, the poly(HEMA-co-3APBA)/RosA MP, and the poly(HEMA-co-3APBA-co-MAPTAC)/RosA MP (ns, not significant; ^*^P < 0.05; ^**^P < 0.01).

To evaluate the skin-piercing capability of the RosA-MP, compression force was directly applied to the microneedle array. Representative force–displacement curves for the B-MP and RosA-MP are presented in Fig. 3A. The corresponding fracture force and fracture energy per microneedle were calculated and are shown in Fig. 3B and C, respectively. Compared to the B-MP, the strength and toughness of a single microneedle in the RosA-MP increased by approximately 44.4% (*P* < 0.05) and 100% (*P* < 0.01), reaching values of 0.13 N and 0.53 mJ, respectively. To further investigate the hardness and modulus of the RosA-MP microneedles, DSI tests were performed. However, for these tests, force was applied directly to bulk samples: poly(HEMA-co-3APBA-MAPTAC) for the B-MP and poly(HEMA-co-3APBA-co-MAPTAC)/RosA for the RosA-MP. Figure 3D shows the representative force–displacement curves, and the corresponding calculated hardness and modulus values are displayed in Fig. 3E and F, respectively. The hardness and modulus of the RosA-MP were 100% (*P* < 0.01) and 40% higher than those of the B-MP (though the modulus increase was not statistically significant), reaching 0.12 and 2.45 GPa, respectively. Figure S4 shows H&E staining images of skin tissues following piercing by the B-MP and RosA-MP. The RosA-MP group exhibited reduced tissue disorganization and greater penetration depth compared to the B-MP group. These results indicate that incorporating RosA molecules into the B-MP significantly enhanced the hardness, strength, and toughness of the resulting RosA-MP, thereby conferring superior skin-piercing capability with minimal tissue damage. ‌This enhancement likely arises from phenylboronic acid ester bond-mediated crosslinks within the RosA-MP, which strengthen the polymer network.

To investigate the release kinetics and duration of RosA from the RosA-MP, we analyzed cumulative release profiles in PBS under 3 different pH conditions (5.5, 7.4, and 9.3) using 7 classical drug release models (Fig. 3G and H). The results showed‌ a pH-dependent release pattern: RosA exhibited prolonged release at pH 5.5 (14 days), ‌whereas‌ the duration ‌shortened‌ to 2 days (pH 7.4) and 1 day (pH 9.3) (Fig. 3G). ‌This behavior correlates with‌ RosA’s conformational transitions in neutral/alkaline environments [36]. Notably‌, the release rates at pH 7.4 and 5.5 ‌significantly exceeded‌ those at pH 9.3 (Fig. 3H), ‌a phenomenon attributed to‌ the pH-sensitive phenylboronic acid ester bond: ‌In acidic/neutral conditions (pH < 8.2)‌, the bond adopts a planar structure with high tension, ‌promoting hydrolysis‌; ‌in alkaline conditions (pH > 8.2)‌, it stabilizes as a tetrahedron, ‌inhibiting cleavage [45,46]. ‌These findings suggested‌ that RosA release was mediated by pH-triggered bond breakage followed by diffusion.

To validate the release mechanism, 7 kinetic models (Zero-order, First-order, Higuchi, Hixson–Crowell, Korsmeyer–Peppas, Kopcha, and Weibull) were fitted to the release curves. Six models (excluding Weibull) exhibited satisfactory convergence‌ (Fig. 3I and J and Figs. S5 to S8), with the Korsmeyer–Peppas and Kopcha models showing superior fit (Fig. 3I and J). Analysis of fitted parameters (Table 2) revealed Fickian diffusion-dominated release‌, evidenced by ‌a Korsmeyer–Peppas exponent *n* < 0.5‌ and ‌Kopcha *A*/*B* ratio >> 1‌. Notably, the *A*/*B* ratio at pH 9.3 ‌was significantly lower‌ than at pH 5.5/7.4, confirming the dual mechanism: ‌pH-dependent phenylboronic acid ester bond cleavage‌ followed by diffusion. To demonstrate MAPTAC’s role in hygroscopic swelling and release acceleration‌, swelling ratio–time curves (Fig. 3K) and cumulative release profiles (Fig. 3L) of the PHEMA/RosA, the poly(HEMA-co-3APBA)/RosA, and the poly(HEMA-co-3APBA-MAPTAC)/RosA were compared. ‌Equilibrium swelling ratios‌ (Tables S2 and S7) further supported that MAPTAC moieties ‌enhanced osmotic pressure‌ within the poly(HEMA-co-3APBA-MAPTAC) matrix, ‌thereby accelerating RosA release‌ and potentially reducing therapeutic response time in tendinopathy.

**Table 2.** Mathematical modeling and related parameters based on release data

	Korsmeyer–Peppas model			Kopcha model		
pH	5.5	7.4	9.3	5.5	7.4	9.3
*R* ^2^	0.999	0.994	0.995	0.999	0.994	0.998
*n*	0.478	0.484	0.415	–	–	–
*A*/*B*	–	–	–	78.503	82.141	19.480

### In vitro evaluation of the capability in inducing M_2_ M_φ_ polarization of the RosA-MP

Biosafety testing of the RosA-MP was performed prior to applying it to biological experiments. The results of the CCK-8 assay revealed that the relative viability of Raw 264.7 cells cocultured with the RosA-MP for 24, 48, or 72 h remained above 90% (Fig. S9A). Furthermore, no significant hemolysis was observed in the RosA-MP group (10 to 100 mg/ml) compared to the saline group (Fig. S9B and C). To provide a more comprehensive assessment of biocompatibility, we additionally performed live/dead cell staining after 48 and 72 h of the RosA-MP exposure. As shown in Fig. S9D and E, the vast majority of cells maintained robust viability as evidenced by intense green fluorescence, while only rare dead cells (red fluorescence) were detected—comparable to the negative control groups. The above results proved the excellent biocompatibility of the RosA-MP.

**Fig. 4.**
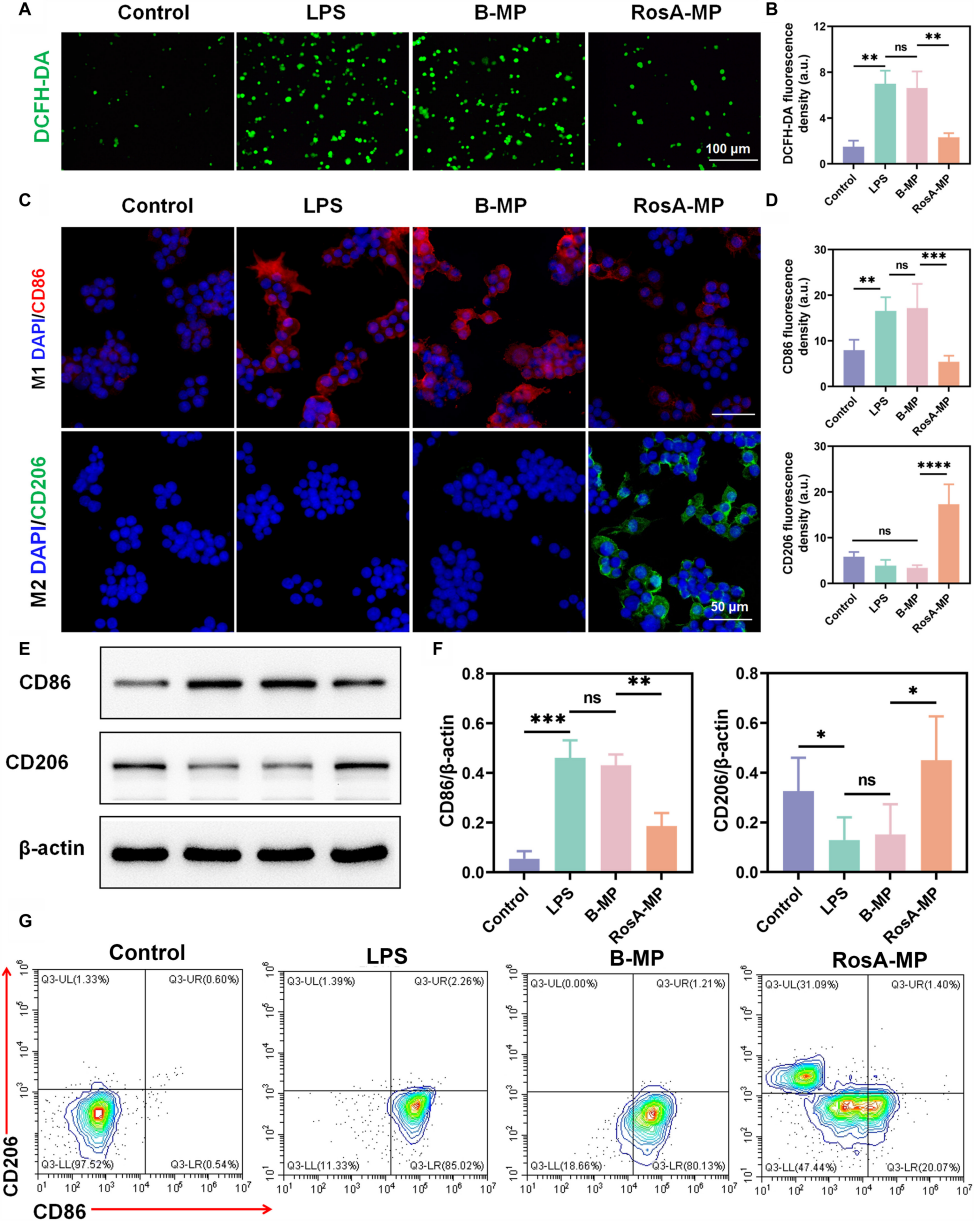
In vitro assessment of the ROS scavenging and immunomodulatory effects of the RosA-MP. (A) Immunofluorescence images showing ROS levels in Raw 264.7 cells after 3 days of different treatments. (B) Quantitative analysis of DCFH-DA (ROS) fluorescence intensity. (C) Immunofluorescence images of CD86 (red), CD206 (green), and nuclei (blue) in Raw 264.7 cells after 3 days of different treatments. (D) Quantification analysis of CD86 and CD206 fluorescence intensity. (E) Western blot analysis of CD86 and CD206 expression in Raw 264.7 cells after 3 days of different treatments. (F) Quantitative comparison of CD86 and CD206 expression levels based on Western blot data. (G) Flow cytometry analysis of the M_1_ M_φ_ marker CD86 and the M_2_ M_φ_ marker CD206 in Raw 264.7 cells after different treatments for 3 days (*n* = 5, ns, not significant; ^*^P < 0.05; ^**^P < 0.01; ^***^P < 0.005; ^****^P < 0.001).

Highly plastic M_φ_ play a key role in the inflammatory response and tissue healing in tendinopathy [47]. Inhibiting M_1_ M_φ_ polarization while promoting M_2_ M_φ_ polarization is critical for tendinopathy therapy [26]. To investigate the immunomodulatory function of the RosA-MP in inducing M_1_-to-M_2_ transition of M_φ_, pristine M_0_ M_φ_ were subjected to 4 different treatments: no treatment (blank control group), adding LPS to mimic a pro-inflammatory microenvironment (LPS group), adding LPS together with the B-MP extract (the B-MP group), and adding LPS together with the RosA-MP extract (the RosA-MP group). At the tendinopathy site, sustainedly activated M_1_ M_φ_ produce excessive ROS, which in turn increase the release of pro-inflammatory factors. Excessive ROS and pro-inflammatory factors disrupt redox homeostasis in tendon cells, tendon progenitor cells, etc., adversely affecting their bioactivity and function. As shown in Fig. 4A and B, intracellular ROS levels in M_φ_ were measured using a DCFH-DA fluorescent probe and visualized via fluorescence imaging. Compared to the control group, LPS treatment significantly increased ROS levels in M_φ_, while the RosA-MP group exhibited a marked reduction in intracellular ROS. In contrast, no significant differences were found between the B-MP and LPS groups.

To investigate the immunoregulatory effects of the RosA-MP on inducing M_2_ M_φ_ polarization, the expression levels of the M_1_ marker CD86 and the M_2_ marker CD206 were analyzed using immunofluorescence staining (Fig. 4C). Semi-quantitative analyses revealed that the LPS group exhibited the highest expression level of CD86 (Fig. 4D), indicating that LPS significantly promoted M_1_ polarization of M_φ_. In contrast, the expression levels of CD86 in the RosA-MP group were significantly reduced compared to the LPS group, suggesting that the RosA released from the RosA-MP effectively inhibited M_1_ polarization. However, the B-MP did not show significant effect on CD86 expression. Additionally, the expression level of CD206 was also the highest in the RosA-MP group, with no significant differences observed among the other 3 groups. Western blot analysis further confirmed these findings, showing that Raw 264.7 cells in the RosA-MP group, compared to the other groups, exhibited higher CD206 expression and lower CD86 expression (Fig. 4E and F). Flow cytometry results also demonstrated that the RosA-MP treatment significantly induced M_1_-to-M_2_ transition of M_φ_ (Fig. 4G), with most cells expressing CD206 while very few cells expressing CD86. To further investigate the influence of the RosA-MP treatment on M_φ_ polarization, ELISA was used to measure secretion levels of cytokines. Notably, the pro-inflammatory marker IL-6, which showed increased expression level following LPS stimulation, was greatly decreased by the RosA-MP treatment (Fig. S10). In contrast, the B-MP treatment did not significantly influence IL-6 secretion. Additionally, the RosA-MP treatment significantly facilitated the secretion of anti-inflammatory cytokine IL-10.

To strengthen the physiological relevance of our findings, we further validated the polarization effect of the RosA-MP using primary mouse BMDMs isolated from mice. Immunofluorescence analysis revealed that Arg1 expression in BMDMs was markedly enhanced in both the RosA-MP and IL-4 groups compared to M_0_ (untreated control)- and LPS (M_1_)-treated groups. Gene expression analysis by qPCR further confirmed these observations: RosA-MP significantly up-regulates the expression of M_2_-associated markers Arg1 and TGF-β compared to both the LPS-treated and untreated control groups, although the effect size is slightly lower than that of the IL-4 group, while M_1_ markers iNOS and TNF-α were significantly suppressed relative to LPS-treated controls (Fig. S11).

In sum, the above results proved that the RosA-MP could simultaneously scavenge excessive intracellular ROS in M_φ_ and induce the transition of M_φ_ from an M_1_ phenotype to an M_2_ phenotype in a simulated tendinopathy microenvironment.

### Investigation of the rationale behind the RosA-MP inducing M_2_ M_φ_ polarization through RNA sequencing and bioinformatic data analysis

**Fig. 5.**
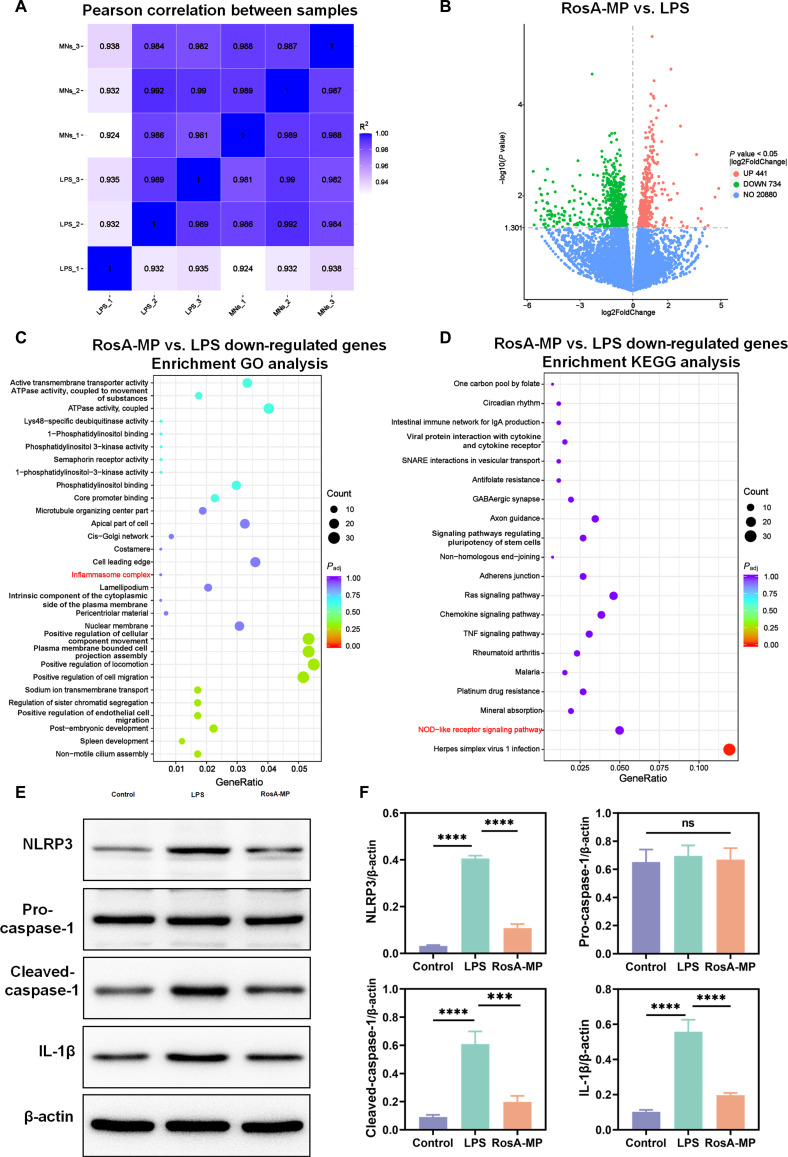
Mechanisms of the RosA-MP in regulating M_φ_ polarization. (A) Heatmap of Pearson correlation among samples. (B) Volcano plot of DEGs between the RosA-MP and LPS groups. (C) GO analysis of up-regulated DEGs in Raw 264.7 cells treated with the RosA-MP versus LPS, with M_φ_ polarization-related pathways highlighted in red. (D) KEGG enrichment of up-regulated DEGs, highlighting pathways related to M_φ_ polarization in red. (E) Western blot analysis of NLRP3, Pro-caspase-1, Cleaved caspase-1, and IL-1β expression in Raw 264.7 cells treated with the RosA-MP and LPS. (F) Quantification of NLRP3, Pro-caspase-1, Cleaved caspase-1, and IL-1β protein expression based on Western blot analysis (*n* = 3, ns, not significant; ^***^P < 0.005; ^****^P < 0.001).

To further elucidate the mechanisms behind M_φ_ polarization modulated by the RosA-MP, transcriptomic mRNA-seq was conducted to investigate its overall impact on Raw 264.7 cells. Sample stability was evaluated using Pearson correlation analysis, with most correlation coefficients falling within an acceptable range (*R*^2^ > 0.95) (Fig. 5A). A volcano plot analysis identified 441 up-regulated and 734 down-regulated genes following the RosA-MP treatment compared to the LPS group (Fig. 5B). GO annotation revealed that the RosA-MP treatment suppressed M_φ_ inflammatory complexes, particularly inflammasomes (Fig. 5C). KEGG enrichment analysis further indicated significant down-regulation of the TNF signaling pathway, the NOD-like receptor (NLR) signaling pathway, and the chemokine signaling pathway in M_φ_ after treatment with the RosA-MP (Fig. 5D).

By integrating the GO and KEGG results, we focused on how the RosA-MP affected the formation of NOD-like receptor pyrin domain-containing (NLRP) inflammasomes in M_φ_. Within the NLRP family, the NLRP3 inflammasome is the most extensively studied. This multiprotein complex, predominantly expressed in monocytes and M_φ_, serves as a key component of innate immunity [13]. It plays a crucial role in pathogen clearance by recognizing pathogen-associated molecular patterns (PAMPs) and damage-associated molecular patterns (DAMPs). Upon activation by various endogenous or exogenous stimuli, the NLRP3 inflammasome facilitates the cleavage of pro-caspase-1 into its active form, resulting in the release of mature IL-1β and the recruitment of other immune cells, thereby driving systemic inflammation. Recent studies have increasingly linked NLRP3 inflammasome activation to an imbalance in M_1_/M_2_ M_φ_ polarization. For example, silencing NLRP3 or caspase-1 has been shown to promote M_2_ polarization in microglial cells, alleviating Alzheimer’s disease. Likewise, during tendinopathy progression, adverse stimuli activate the NLRP3 inflammasome, triggering M_1_ M_φ_ polarization and IL-1β production. Emerging evidence suggests that the abnormal activation of the NLRP3 inflammasome is closely associated with the pathogenesis of tendinopathy [48–50]. To verify the effects of the RosA-MP on key proteins within the NLRP3 inflammasome, Western blot analyses were conducted. As shown in Fig. 5E and F, the protein levels of NLRP3, cleaved caspase-1, and IL-1β were significantly elevated in the LPS group compared to the control group. However, the RosA-MP treatment significantly down-regulated the expression levels of these proteins, suggesting that the suppression of the NLRP3 inflammasome was a potential mechanism by which the RosA-MP regulated M_φ_ polarization. To further validate whether suppression of the NLRP3 inflammasome directly contributes to M_2_ M_φ_ polarization, we performed additional loss-of-function experiments. Pharmacological inhibition of NLRP3 with MCC950 was employed in BMDM. Notably, MCC950 treatment promoted M_2_ M_φ_ marker (CD206) expression and reduced M_1_ marker (CD86), similar to the effects observed with the RosA-MP treatment (Fig. S12), supporting the pivotal role of NLRP3 inflammasome suppression in mediating the pro-M_2_ effects of the RosA-MP.

### In vivo investigation of the capability of the RosA-MP in alleviating collagenase-induced tendinopathy

**Fig. 6.**
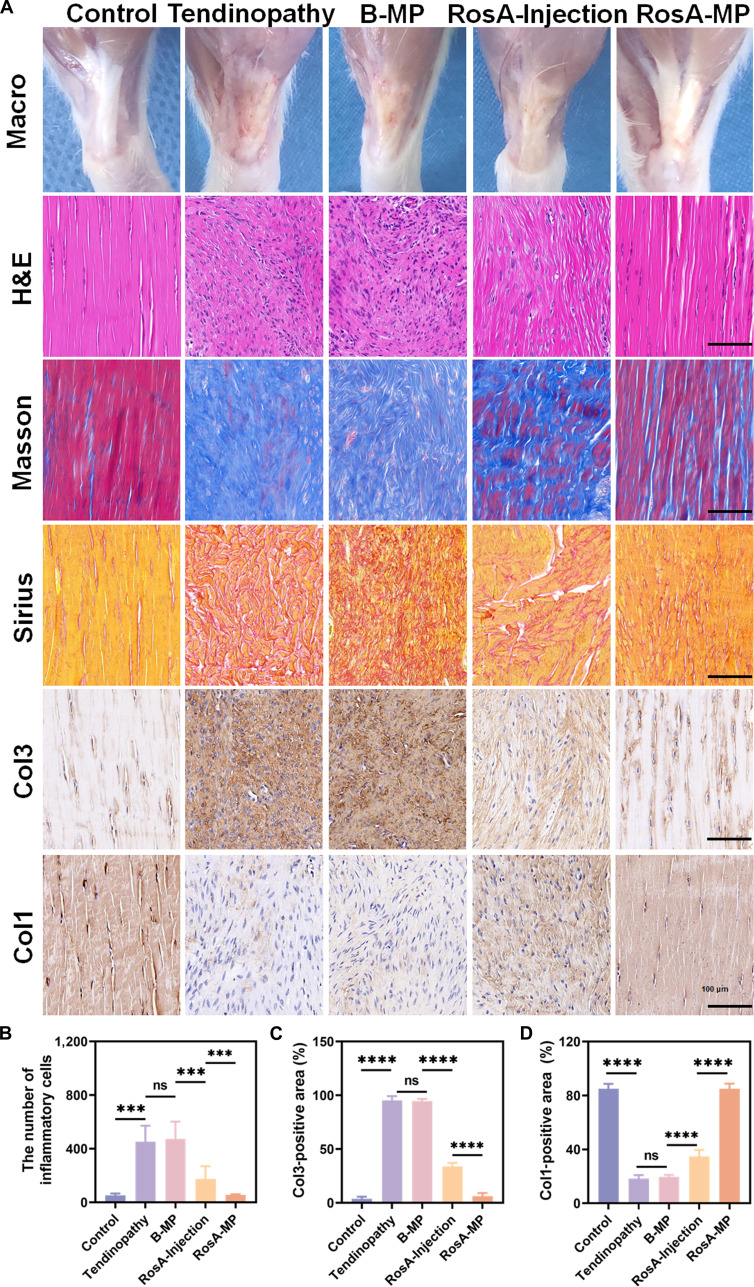
In vivo evaluation of the RosA-MP in promoting tendinopathy recovery. (A) Representative macroscopic images, H&E staining, Masson staining, Sirius red staining, and immunohistochemistry of tendons after 4 weeks of different treatments across 5 groups. (B) Quantitative analysis of inflammatory cell counts in the 5 groups. (C and D) Proportions of Col1-positive and Col3-positive areas in the 5 groups. (*n* = 5, ns, not significant; ^***^P < 0.005; ^****^P < 0.001).

To assess the therapeutic effects of the RosA-MP on tendinopathy, in situ administration was performed following collagenase I injection. Four weeks post-operation, the animals were sacrificed, and tendon healing was analyzed through macroscopic and histological techniques, including H&E, Masson, Sirius red staining, and immunohistochemistry. Macroscopically, Achilles tendons in the tendinopathy group appeared swollen, rough, and adhered to surrounding tissues. In contrast, the RosA-MP treatment restored the tendon’s smoothness and normal volume (Fig. 6A). H&E staining showed disorganized collagen fibers and significant inflammatory cell infiltration in the tendinopathy group. However, RosA injection improved collagen alignment, which was even more pronounced in the RosA-MP group (Fig. 6A). Quantitative analysis confirmed that inflammatory cell infiltration was significantly reduced in the RosA-MP group, nearing levels observed in healthy tendons (Fig. 6B). Masson staining indicated collagenase-induced swelling, featuring thick, disorganized collagen fibers (blue-stained) and reduced extracellular matrix (red-stained). These pathological changes were effectively reversed by the RosA-MP treatment (Fig. 6A). ImageJ analysis of Masson trichrome-stained sections demonstrated that the collagen alignment neatness (coherency value) in the tendinopathy group was significantly decreased compared to the healthy group, suggesting profound disruption of tendon matrix structure (Fig. S13). Notably, the RosA-MP treatment substantially improved the collagen fiber alignment. Additionally, Sirius red staining revealed that tendons in the RosA-MP group displayed a bright red, continuous, and well-aligned morphology, in contrast to the irregular orientation seen in the tendinopathy group (Fig. 6A).

Immunohistochemical analysis demonstrated clear changes in Col1a and Col3 expression. Healthy tendons exhibited high levels of neatly arranged Col1a and minimal Col3, with visible nuclei. In the tendinopathy and B-MP groups, Col3 expression was significantly elevated, while Col1a levels were reduced. The RosA-MP treatment resulted in superior tendon repair compared to the RosA injection, as evidenced by a greater reduction in the Col3-positive area (Fig. 6A and C) and near-normal Col1-positive area levels (Fig. 6A and D). Although the RosA injection improved Col1 expression compared to the tendinopathy group, the levels were still lower than those in the RosA-MP group. Since the strength of the Achilles tendon decreases after injury, we conducted mechanical tests on the Achilles tendon to investigate the effects of the RosA-MP treatment on the strength of the Achilles tendon. As shown in Fig. S14, the tendinopathy and the B-MP groups exhibited significant reductions in maximum force, stiffness, elastic modulus, yield stress, and failure strain compared to the healthy control. Importantly, the RosA-MP treatment remarkably enhanced all biomechanical outcomes. These parameters were all significantly improved compared with the tendinopathy and the B-MP groups.

In summary, while RosA injection provided partial repair of damaged Achilles tendons, the RosA-MP significantly facilitates tendon healing, restoring closer-to-normal structure and function.

### In vivo investigation of the antioxidant and immunomodulatory effects of the RosA-MP

**Fig. 7.**
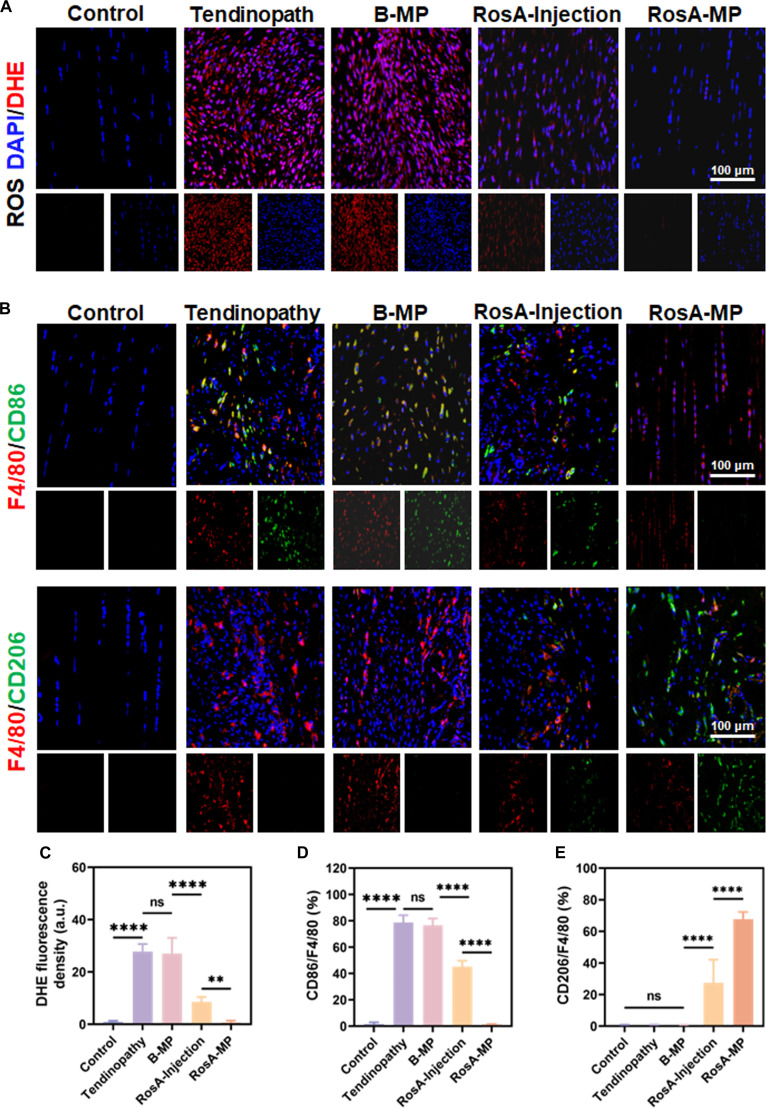
In vivo evaluation of the antioxidant and immunomodulatory effects of the RosA-MP. (A) Immunofluorescence images of DHE-stained Achilles tendon tissues after 4 weeks of different treatments from tendinopathy mice in the 5 groups. (B) Immunofluorescence of anti-F4/80 (red) and anti-CD86/CD206 (green) in tendon sections in the 5 groups. (C) Corresponding statistical analysis of the fluorescence intensities of DHE. (D and E) Ratio of CD86^+^ cells to F4/80^+^ cells (CD86/F4/80) and CD206^+^ cells to F4/80+ cells (CD206/F4/80) (*n* = 5, ns, not significant; ^**^P < 0.01; ^****^P < 0.001).

DHE, a widely utilized redox-sensitive fluorescent probe, is commonly employed for detecting superoxide and hydrogen peroxide. After 4 weeks of various treatments, DHE staining was used to evaluate superoxide production in tendons (Fig. 7A and C). The tendinopathy group displayed intense DHE red fluorescence at the site of tendon injury, indicating significantly elevated ROS levels compared to the control group. The RosA-MP treatment effectively reduced ROS levels to nearly undetectable levels in the tendons. While the RosA injection alone also lowered ROS production, the reduction was less pronounced compared to the RosA-MP group. Additionally, the B-MP treatment had minimal impact on ROS levels in the tendinopathy model.

To investigate whether the RosA-MP-enhanced tendon repair was linked to M_φ_ polarization in vivo, we analyzed M_φ_ infiltration within Achilles tendons across different groups. Immunofluorescence staining revealed extensive infiltration of CD86-positive M_φ_ (pro-inflammatory M_1_ subtype) in the tendinopathy group, which was significantly reduced following the RosA-MP treatment (Fig. 7B and D). Moreover, The RosA-MP treatment led to a notable increase in CD206-positive M_φ_ (anti-inflammatory M_2_ subtype) compared to the tendinopathy and RosA injection groups (Fig. 7B and E). These results indicated that the RosA-MP treatment effectively promoted the polarization of M_φ_ from a pro-inflammatory M_1_ phenotype to a pro-reparative M_2_ phenotype, thereby facilitating tendon healing.

### In vivo organ toxicity investigation after the RosA-MP treatment

After 4 weeks of treatment with the RosA-MP, the heart, liver, spleen, lungs, and kidneys of the rats were collected and analyzed via H&E staining, revealing no evidence of significant toxicity (Fig. S15). Myocardial tissues displayed well-organized muscle fibers, and liver tissues exhibited normal hepatocyte morphology with clear, distinct boundaries. The glomeruli appeared intact with normal size and shape, while no abnormal inflammatory cell infiltration was observed in any of the examined organs. Combined with the results of in vitro cytotoxicity and hemocompatibility assays, these findings confirmed the excellent biocompatibility of the RosA-MP, underscoring its suitability for tendinopathy treatment.

## Conclusion

Tendinopathy is a high-morbidity tendon disease that significantly impairs patients’ quality of life. Recent research implicates M_1_ M_φ_ infiltration as a key driver of tendinopathy, simultaneously exacerbating inflammation and ROS accumulation, and inhibiting tendon healing. As tendinopathy therapy requires sustained intervention, persistently inducing in situ M_1_-to-M_2_ M_φ_ transition represents a rational therapeutic strategy. However, current approaches for achieving localized M_2_ M_φ_ polarization at the tendinopathy site lack sufficient efficacy for practical applications. While MP-mediated in situ drug delivery shows promise, existing MP designs often underestimate the critical role of M_φ_ and immunomodulation in treating tendinopathy.

This work presents a RosA-loaded MP (RosA-MP) designed for tendinopathy treatment. The RosA-MP features adaptive structural properties, superior skin penetration capability, and sustained RosA release, enabling simultaneous in situ M_2_ M_φ_ polarization and ROS scavenging at the lesion site. The RosA-MP was fabricated by first synthesizing a poly(HEMA-co-3APBA-co-MAPTAC) microneedle array via bulk copolymerization of HEMA, 3APBA, and MAPTAC monomers. This array was covalently bonded to a flexible filter paper backing. RosA was then incorporated by equilibrating the microneedle array in an ethanolic RosA solution, followed by solvent evaporation.

FTIR, XRD, and XPS analyses confirmed homogeneous dispersion of RosA within the RosA-MP and formation of phenylboronic acid ester bonds between the 3APBA moieties and RosA molecules. TG determined the RosA loading to be 7.16% (w/w). differential scanning calorimetry (DCS) indicated that the phenylboronic acid ester bonds stabilized RosA. Compressing and DSI tests demonstrated that these bonds significantly increased the microneedle hardness (0.12 GPa), fracture strength (0.13 N), and fracture energy (0.53 mJ) compared to the blank MP (B-MP). Consequently, the RosA-MP caused less tissue damage than the B-MP during skin penetration. In vitro release studies showed sustained RosA release from the RosA-MP for over 24 h. Release kinetics modeling and parameter analysis indicated that release primarily involved cleavage of the phenylboronic acid ester bonds followed by Fickian diffusion. Swelling assays and supplementary release studies confirmed that MAPTAC moieties accelerated hygroscopic swelling of the RosA-MP, thereby accelerating RosA release.

In vitro Raw 264.7 cell assays using the RosA-MP extracts verified potent ROS scavenging and M_1_-to-M_2_ M_φ_ polarization capabilities. RNA sequencing and bioinformatic analysis revealed that this immunomodulation involves suppression of the NLRP3 inflammasome. Notably, mRNA-seq in this study was performed specifically on Raw 264.7 cells to provide a clear, cell-type-specific view of the transcriptional responses and signaling pathways underlying M_φ_ polarization. Nevertheless, we fully recognize the value of tissue-level, and even single-cell transcriptomic analyses in future studies to illuminate the in vivo mechanisms underlying the antioxidant and immunomodulatory actions of the RosA-MP and how these effects orchestrate tendon repair. Inclusion of such in vivo omics approaches will be critical to further elucidate the complex, synergistic interactions that contribute to enhanced tendon healing following the RosA-MP treatment. The therapeutic efficacy of the RosA-MP was evaluated in a rat model of Col1-induced tendinopathy. Macroscopic and histological analyses demonstrated significant reversal of tendinopathic changes. In vivo antioxidant and immunomodulation assays further confirmed the dual therapeutic mechanism: simultaneous in situ M_2_ M_φ_ polarization and ROS scavenging at the tendinopathy site.

Future studies should prioritize the following: (a) long-term efficacy (6 to 12 months) to assess sustained effects and M_φ_ exhaustion risks; (b) diverse animal models (mechanical/chronic/metabolic) beyond Col1-induced acute inflammation; and (c) translational optimization, including GMP-scale production, large-animal dose trials (equine/porcine), and rehabilitation synergy. These steps will validate RosA-MP’s durability, broad applicability, and clinical readiness while addressing current gaps between preclinical success and human therapies.

## Data Availability

The data that support the findings can be obtained from the corresponding authors on reasonable request.
