# Supplementary material for: Inducing In Situ M2 Macrophage Polarization for Tendinopathy Therapy through Microneedle Patch-Mediated Instant/Sustained Delivery of Rosmarinic Acid
Source: Biomater Res. 2025 Nov 8;29:0264. doi: 10.34133/bmr.0264 (PMC12682951; doi:10.34133/bmr.0264)
Supplement: Supplementary 1 — Figs. S1 to S15 Tables S1 to S7 [file bmr.0264.f1.docx]

**Inducing *in situ* M_2_ Macrophage Polarization for** **Tendinopathy Therapy through Microneedle Array Mediated Local/Sustained Delivery of Rosmarinic Acid**

Zheng Wang^a, b, 1^, Ying Chu^c, d, 1^, Yu Hu^c, d^, Xue Fang^a, b^, Jingyi Du^c, d^, Mingshuang Li^c, d^, Yuxin Zha^c, d^, Jiabing Ran^c, d, *^, Aixi Yu^a, b, *^

^a^ Department of Orthopedics Trauma and Microsurgery, Zhongnan Hospital of Wuhan University, Wuhan, 430000, China

^b^ Hubei Clinical Medical Research Center of Trauma and Microsurgery, Wuhan, 430000, China

^c^ Hubei Key Laboratory of Natural Products Research and Development, China Three Gorges University, Yichang, 443002, China

^d^ College of Biological and Pharmaceutical Sciences, China Three Gorges University, Yichang, 443002, China

^*^Corresponding Authors: Jiabing Ran ([jiabingran@outlook.com](mailto:jiabingran@outlook.com); [ranjiabing@ctgu.edu.cn](mailto:ranjiabing@ctgu.edu.cn)); Aixi Yu ([yuaixi@whu.edu.cn](mailto:yuaixi@whu.edu.cn)).

^1^ contributed equally to this paper

**Table.S1** The initial feeding composition of HEMA, 3APBA, MBAA, MAPTAC, DMPA, TEMED, and RosA

| **Reagent** | **HEMA** | **3APBA** | **MBAA** | **DMPA** | **TEMED** | **MAPTAC** | **RosA** |
| --- | --- | --- | --- | --- | --- | --- | --- |
| Volume  (mL) | **10.00** | **-** | **-** | **-** | **0.03** | **-** | **-** |
| Weight  (mg） | 10728.89 | **6.30** | **2.5** | **21.13** | **-** | **909.80** | **-** |
| Mole number  (mmol) | 82.44 | 0.03 | 0.016 | - | **-** | 4.12 | **-** |
| Concentration (×10^-6^ mol/mL) | - | **-** | **-** | **-** | **-** | **-** | 1.14 |
| Mass/volume concentration (mg/mL) | - | **-** | **-** | **-** | **-** | **-** | **0.41** |


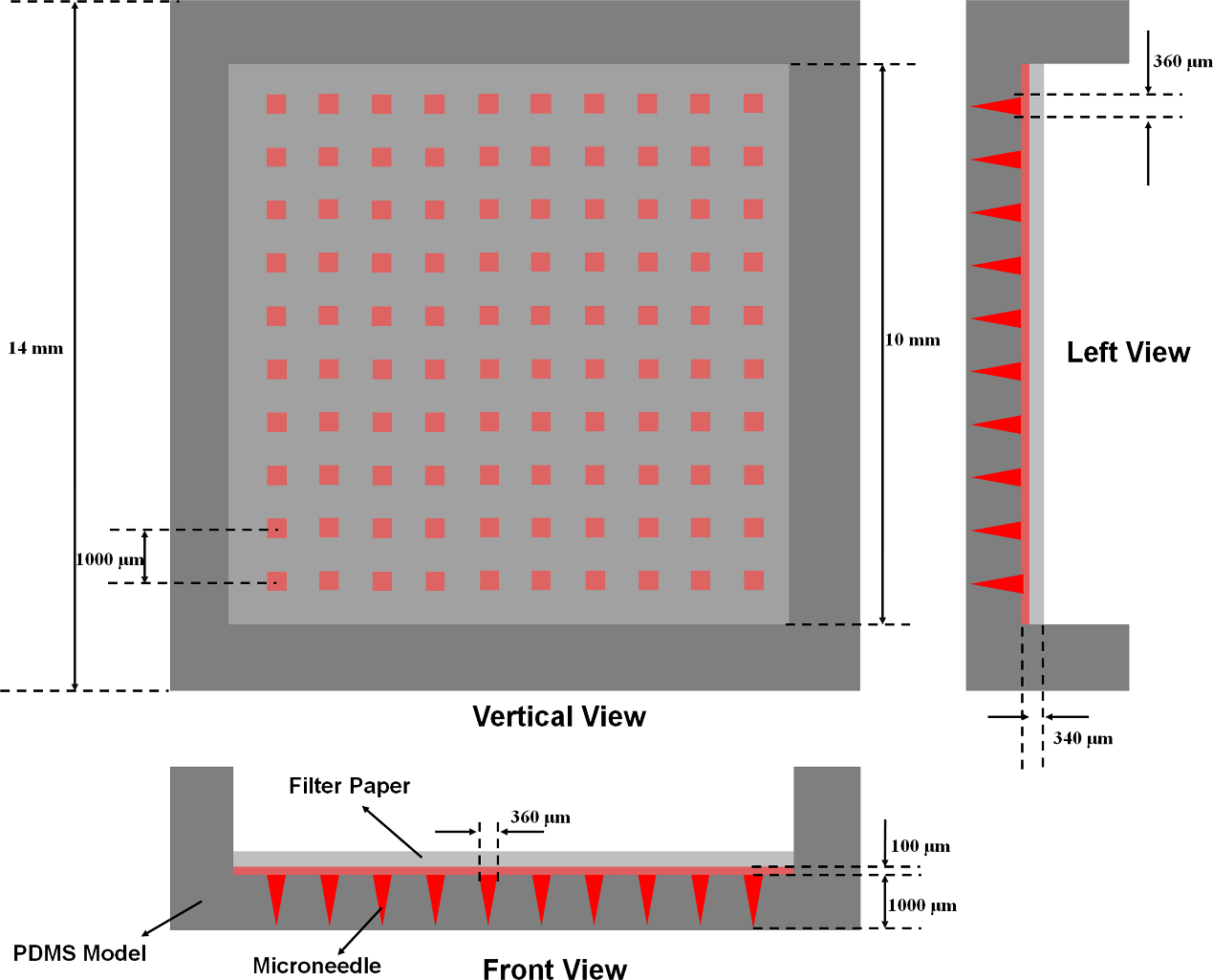


**Fig.S1** Three-view drawing of the RosA-MP

**S1. Swelling behavior of the as-prepared B-MP in PBS and ethanol**

A B-MP was weighed (m_1_) and then soaked in PBS. Until the swelling equilibrium was reached, the swollen B-MP was taken out and weighed (m_2_). The swelling ratio (α) of the B-MP could be calculated using the following equation:

$\alpha=\frac{m_{2}-m_{1}}{m_{1}} \times100\%$ (1)

The swelling ratio (α’) of the B-MP in ethanol could be obtained using the same method. **Table.S2 and Table.S3** show the corresponding swelling data in PBS and ethanol, respectively. Thus, the swelling ratios of the B-MP in PBS (α) and ethanol (α’) were **48****.12 ± 1.55 %** and **70.74 ± 1.50 %**, respectively.

**Table.S2** Corresponding equilibrium swelling data of the B-MP in PBS

| Sample | Before (g) | After (g) | Weight increase (g) | Weight Increase Ratio α (%) |
| --- | --- | --- | --- | --- |
| 1 | 0.4290 | 0.6421 | 0.2131 | 49.67 |
| 2 | 0.3298 | 0.4835 | 0.1537 | 46.60 |
| 3 | 0.3800 | 0.5616 | 0.1816 | 47.78 |

**Table.S3** Corresponding equilibrium swelling data of the B-MP in ethanol

| Sample | Before (g) | After (g) | Weight increase (g) | Weight Increase Ratio α^’^ (%) |
| --- | --- | --- | --- | --- |
| 1 | 0.3752 | 0.6362 | 0.2610 | 69.56 |
| 2 | 0.4237 | 0.7306 | 0.3069 | 72.43 |
| 3 | 0.3265 | 0.5558 | 0.2293 | 70.23 |

**S2. The rationale behind the concentration setting of the ethanol solution of RosA**

The molality of 3APBA in the as-prepared B-MP could be calculated using the following equation:

$m_{3APBA}=\frac{n_{3APBA}}{m_{B-MP}}$ (2)

Here, n_3APBA_ is the mole number of 3APBA and m_B-MP_ is the weight of the B-MP. In this work, the mole number of 3APBA was kept at 1/2500 of that of HEMA. Thus, the m_3APBA_ was approximated to 2.56×10^-6^ mol/g.

As to a B-MP with weight of m_1_, the mole number of 3APBA was around 2.56×10^-6^×m_1_ mol. Here, the mole ratio of 3APBA/RosA of a RosA-MP was kept at 2.5/1. So the mole number of RosA within the Poly(HEMA-co-3APBA-co-MAPTAC)/RosA-MP should be kept at 1.024×10^-6^×m_1_ mol.

According to the swelling ratio of a B-MP in ethanol (70.74 %), we can calculate the weight of ethanol absorbed in the B-MP, 0.7074×m_1_ g. So, the volume of ethanol should be:

$V_{ethanol}=\frac{0.7074 m_{1}}{\rho_{ethanol}}$ (3)

Where ρ_ethanol_ is the density of ethanol, equal to 0.789 g/mL.

Then, we can calculate the concentration of RosA using the following equation:

$c_{RosA}=\frac{1.024\times{10}^{-6}\times m_{1}}{V_{ethanol}}=1.1421\times{10}^{-6} mol/mL$ (4)

So, the weight/volume concentration of the ethanol solution of RosA should be:

$c_{m/V}=c_{RosA}\times360.31\frac{g}{mol}=0.4115 mg/mL$ *(5)*


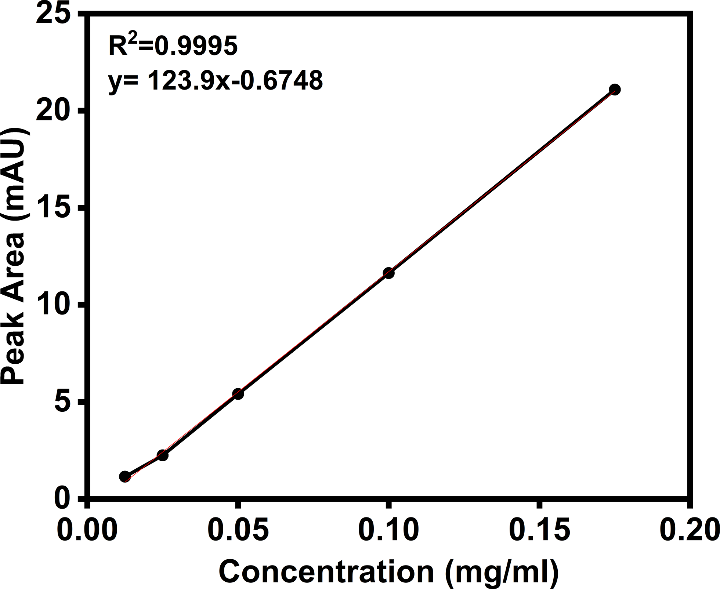


**Fig.S2** Standard curve of RosA plotted through HPLC

**S3. Mathematical Modeling and release kinetics**

1. Zero-order model

$$Q_{t}=Q_{0}+K_{0}t$$

Where Q_t_ is the amount of drug dissolved in time t, Q_0_ is the initial amount of drug in the solution (most times, Q_0_=0) and K_0_ is the zero-order release constant expressed in units of concentration/time.

Application: This relationship is utilized to describe the drug dissolution of several types of modified release pharmaceutical dosage forms, as in the case of some transdermal systems, as well as matrix tablets with low soluble drugs in coated forms, osmotic systems, etc.

1. First-order model

$$\log\left( C \right)=\log\left( C_{0} \right)-kt/2.303$$

Where C_0_ is the initial concentration of drug, k is the first order rate constant, and t is the time.

Application: This relationship is used to describe the drug dissolution in pharmaceutical dosages forms such as those containing water-soluble drugs in porous matrices.

1. Higuchi model

$$Q_{t}=Q_{0}+k_{H}t^{\frac{1}{2}}$$

Where Q_t_ is the cumulative amount of drug release at time t, Q_0_ is the initial amount of drug, k_H_ is the Higuchi constant.

Application: This relationship is used to describe the drug release from an insoluble matrix based on Fickian diffusion. It also can be used to describe the drug dissolution from several types of modified release pharmaceutical dosage forms, as in the case of some transdermal systems and matrix tablets with water soluble drugs.

1. Hixson-Crowell model

$$W_{0}^{1/3}-W_{t}^{\frac{1}{3}}=\kappa t$$

Where W_0_ is the initial amount of drug in the pharmaceutical dosage form, W_t_ is the remaining amount of drug in the pharmaceutical dosage form at time t and κ is a constant incorporating the surface-volume relation.

Application: This expression applies to pharmaceutical dosage form such as tablets, where the dissolution occurs in planes that are parallel to the drug surface if the tablet dimensions diminish proportionally, in such a manner that the initial geometrical form keeps constant all the time.

1. Korsmeyer-Peppas model

$$Q_{t}=K_{kp}t^{n}$$

Where Q_t_ is the cumulative amount of drug release at time t, K_kp_ is the Korsmeyer-Peppas constant, n is the release exponent which defines the drug release mechanism.

Application: This relationship is used to describe drug release from the polymeric system. When the Korsmeyer-Peppas model is used to thin films, the release index n (n = 0.5 or n ≤ 0.5) conforms to Fickian diffusion, while the values of n (0.5＜n＜1) is related to non-Fickian release, meaning that the drug release follows both erosion and diffusion mechanisms. And n = 1 corresponds to the zero-order release which defines the drug release is independent of time.

1. Kopcha model

$$Q_{t}=At^{1/2}+Bt$$

Where A is the diffusion constant and B is the erosional exponent.

Application: If the ratio of A/B is high (A is much greater than B), implying the drug release will follow the diffusion mechanism; and if A/B is low (B is much greater than A), meaning that the polymer erosion or relaxation plays a dominant role in drug release.

1. Weibull model

$M=M_{0}[1-e^{-\frac{{(t-T)}^{b}}{a}}$]

Where M is the amount of drug dissolved as a function of time t. M_0_ is total amount of drug being released. T accounts for the lag time measured as a result of the dissolution process. Parameter a denotes a scale parameter that describes the time dependence, while b describes the shape of the dissolution curve progression.

Application: The Weibull model is more useful for comparing the release profiles of matrix type drug delivery.

**Table. S4** The relevant information of the antibodies for immunofluorescence staining.

|  | **Name** | **Species** | **Manufacturer** | **Catalog Number** |
| --- | --- | --- | --- | --- |
| Primary antibody | CD206 | Rabbit | Abcam, USA | ab64693 |
|  | CD86 | Rabbit | HABIO, China | ER1906-01 |
|  | Arg1 | Rabbit | Goodbio, China | GB11119 |
|  | iNOS | Rabbit | Goodbio, China | GB11285 |
|  | F4/80 | Rabbit | Proteintech, China | 28463-1-AP |
| Secondary antibody | HRP-Goat anti Rabbit | Goat | SeraCare, USA | 5220-0336 |

**Table. S5** The relevant information of the antibodies for western blot.

|  | **Name** | **Species** | **Manufacturer** | **Catalog Number** |
| --- | --- | --- | --- | --- |
| Primary antibodies | CD206 | Rabbit | Proteintech, China | 18704-1-AP |
|  | CD86 | Rabbit | CST, USA | 19589 |
|  | NLRP3 | Rabbit | Proteintech, China | 30109-1-Ap |
|  | Pro-caspase-1 | Rabbit | Abcam, USA | ab179515 |
|  | Cleaved-caspase-1 | Rabbit | Affinity, USA | AF4005 |
|  | IL-1β | Rabbit | Proteintech, China | 26048-1-AP |
|  | β-actin | Rabbit | BIOSS, China | BS-0061R |
| Secondary antibodies | HRP-Goat anti Rabbit | Goat | SeraCare, USA | 5220-0336 |

**Table. S6** The relevant information of the antibodies for immunohistochemical staining.

|  | **Name** | **Species** | **Manufacturer** | **Catalog Number** |
| --- | --- | --- | --- | --- |
| Primary antibody | Col1 | Rabbit | Goodbio, China | GB11022-3 |
|  | Col3 | Rabbit | Goodbio, China | GB111629 |
| Secondary antibody | HRP-Goat anti Rabbit | Goat | SeraCare, USA | 5220-0336 |

**
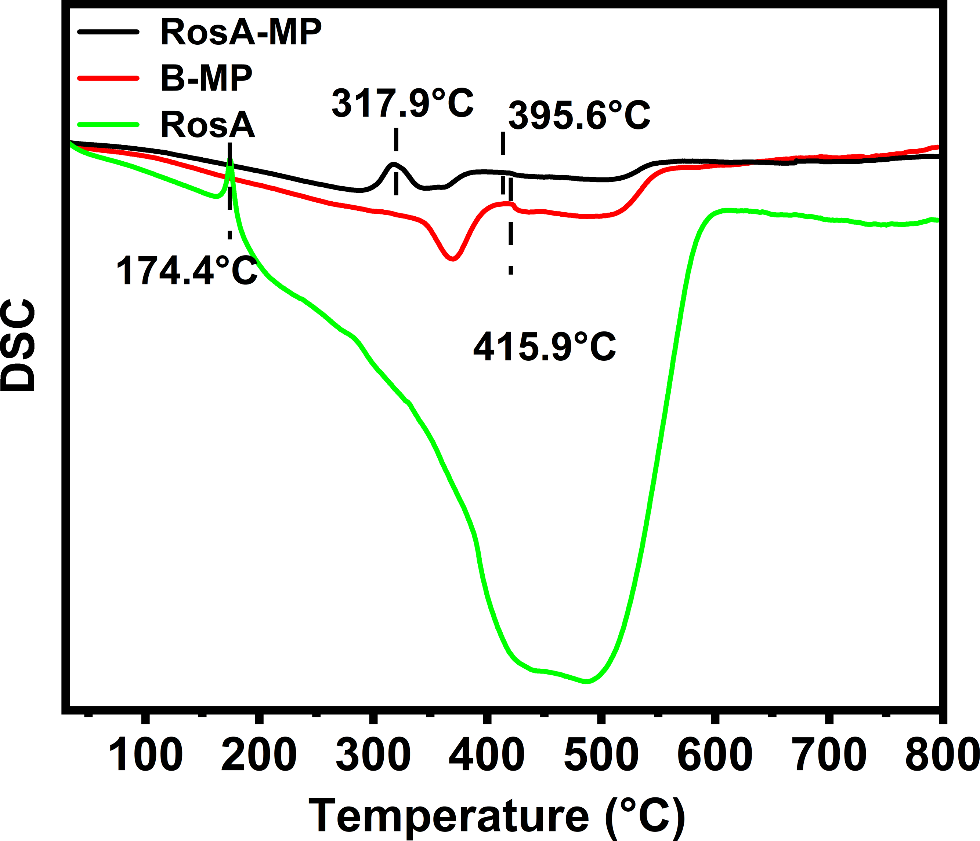
Fig.S3** DSC curves of RosA, the B-MP, and the RosA-MP


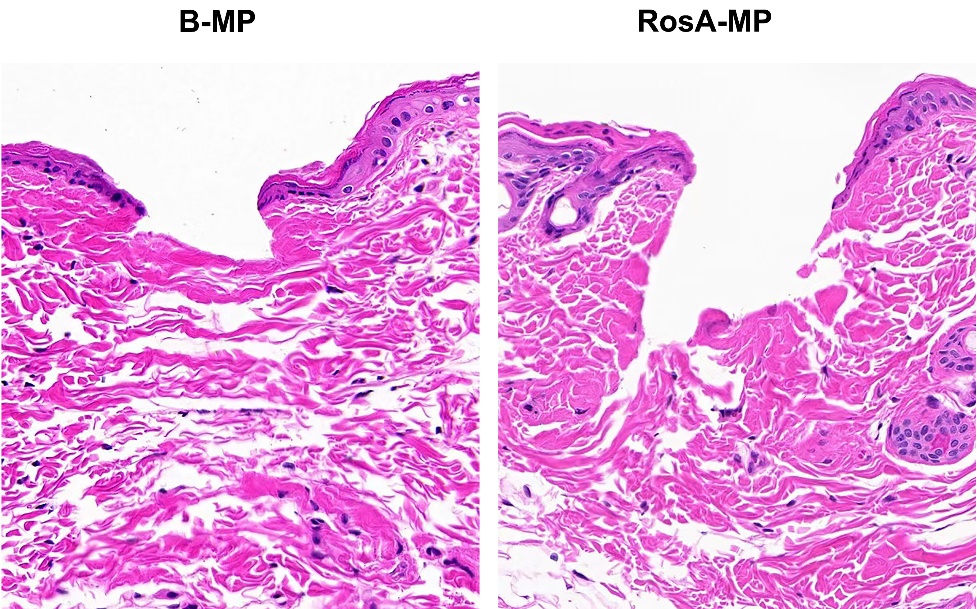


**Fig.S4** H&E staining images of the skin after the B-MP and the RosA-MP puncture

**
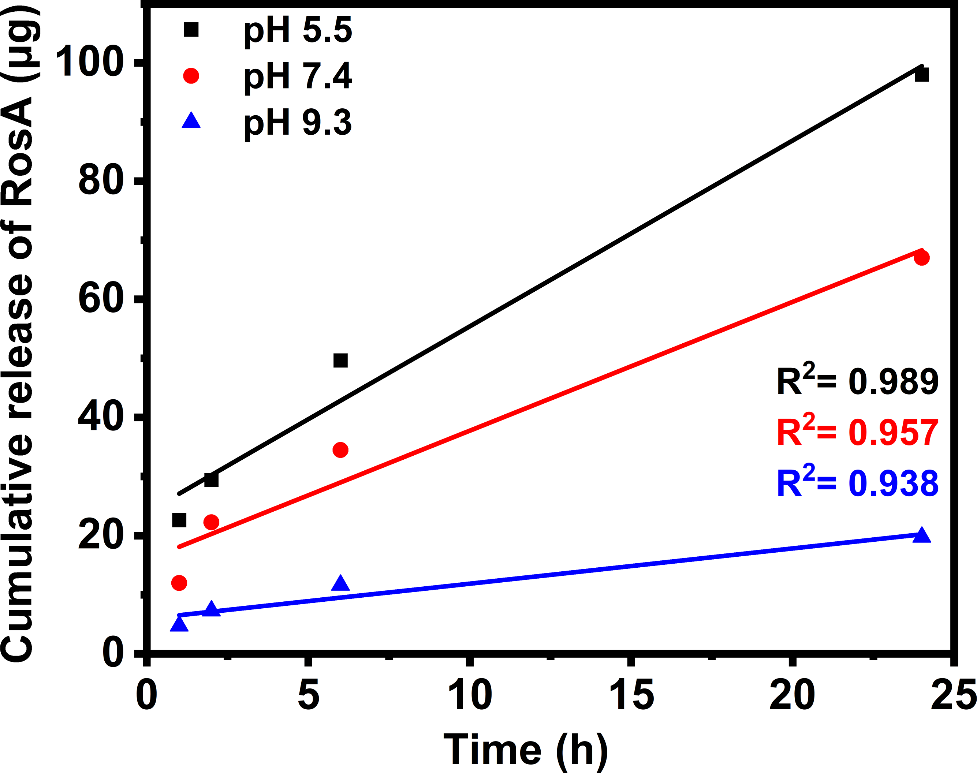
Fig.S5** Cumulative releasing curves of RosA from the RosA-MP fitted using a Zero-order model

**Fig.S6** Cumulative releasing curves of RosA from the RosA-MP fitted using a First-order mode
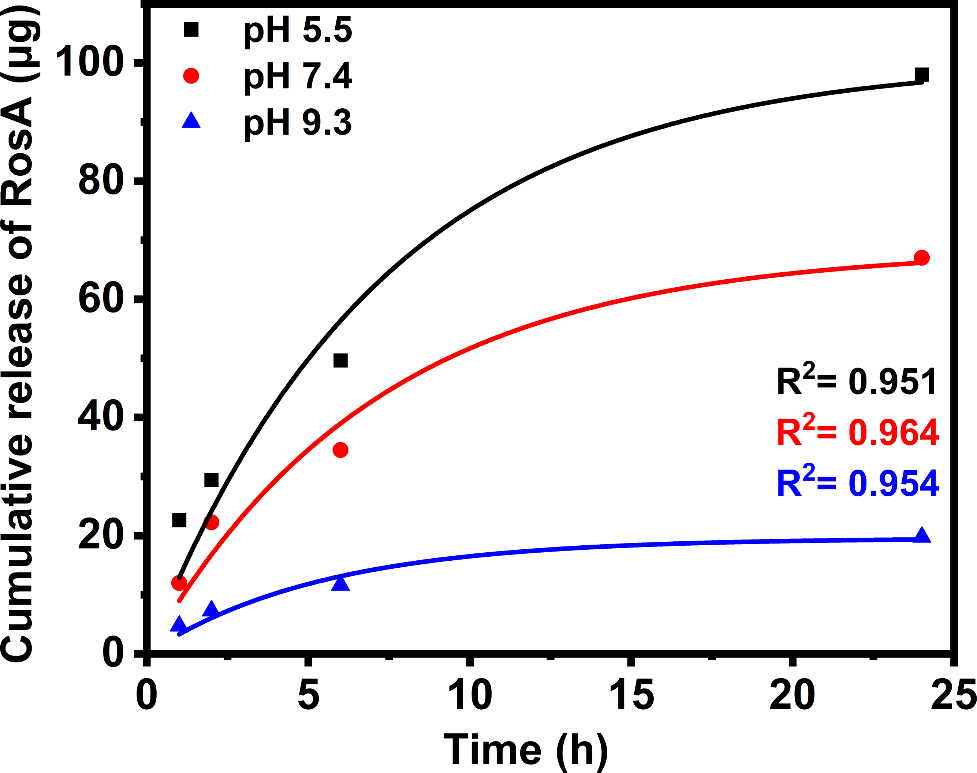
l


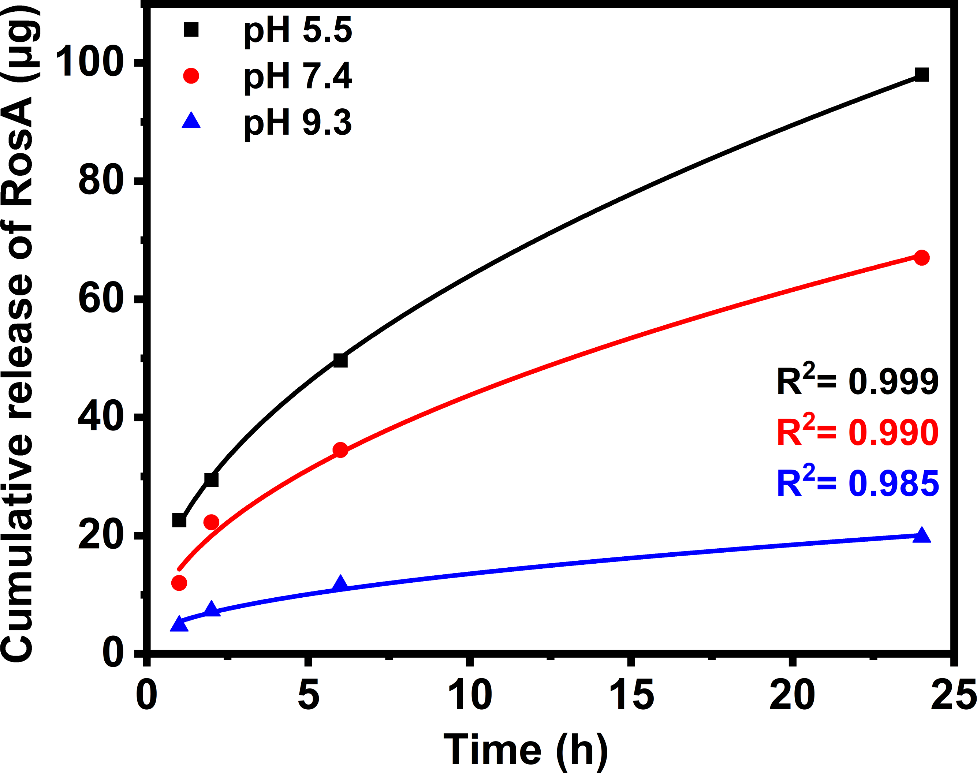
**Fig.S7** Cumulative releasing curves of RosA from the RosA-MP fitted using a Higuchi model


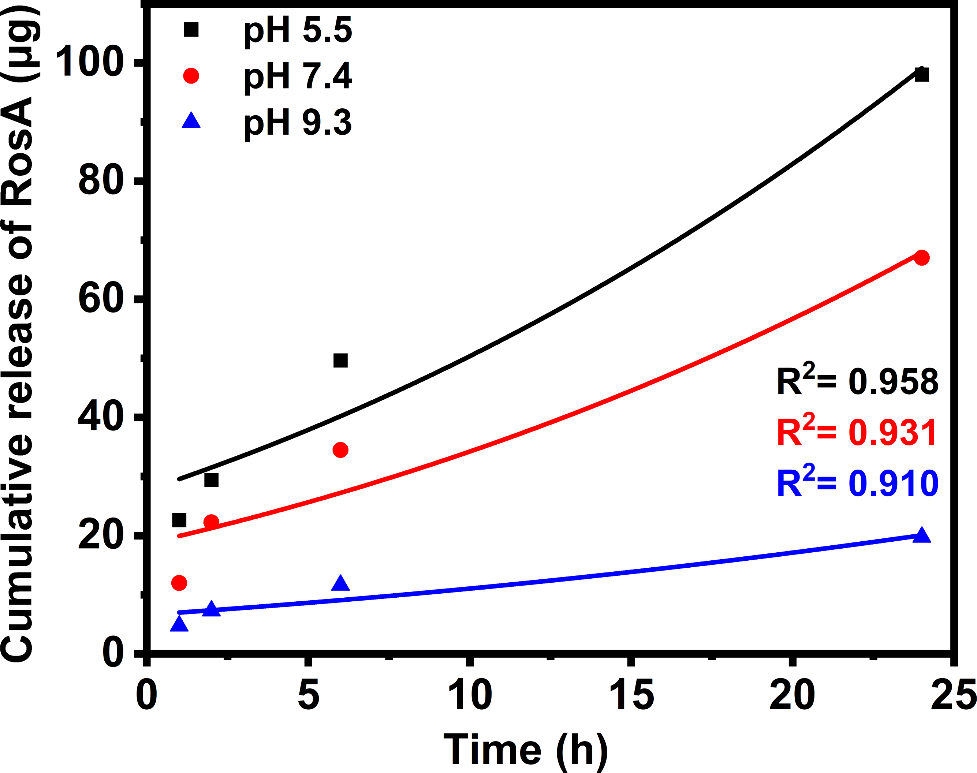


**Fig.S8** Cumulative releasing curves of RosA from the RosA-MP fitted using a Hixson-Crowell model

**Table.S7** Corresponding equilibrium swelling data of the Poly(HEMA-co-3APBA) in PBS

| Sample | Before (g) | After (g) | Weight increase (g) | Weight Increase Ratio α (%) |
| --- | --- | --- | --- | --- |
| 1 | 0.3658 | 0.5102 | 0.1444 | 39.48 |
| 2 | 0.3971 | 0.5530 | 0.1559 | 39.25 |
| 3 | 0.4228 | 0.5774 | 0.1546 | 36.57 |


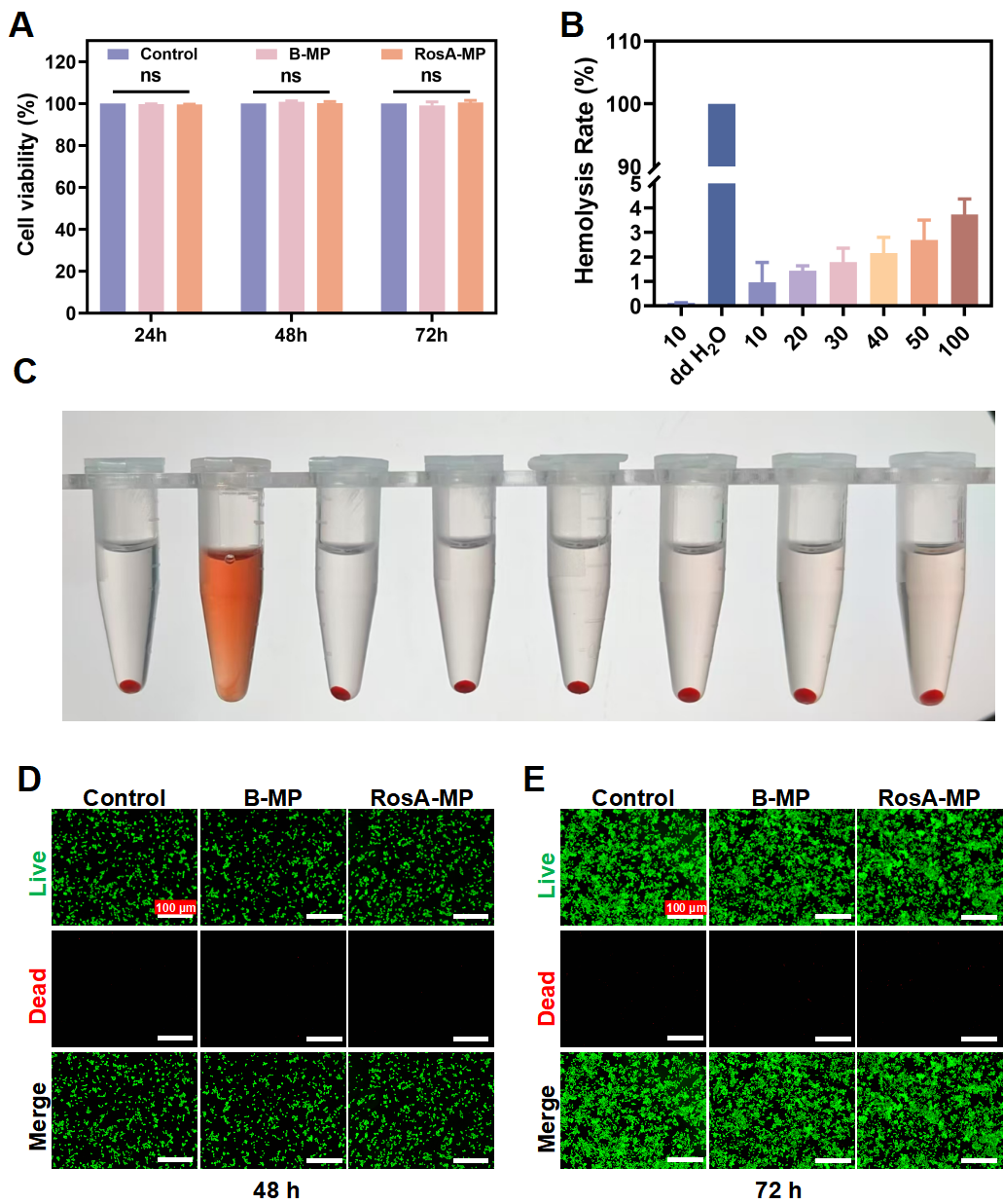


**Fig.S9** Biocompatibility and hemocompatibility evaluation of RosA-MP. (A) CCK-8 assay results for Raw264.7 cells co-cultured with RosA-MP for 24, 48, and 72 hours. (B) Quantitative analysis of hemolysis ratios. (C) Representative images of the hemolysis assay after co-incubation with RosA-MP. (D, E) Live/dead staining of Raw 264.7 cells after incubation with the RosA-MP for 48 h (D) and 72 h (E). Live cells were stained green with calcein-AM, and dead cells were stained red with propidium iodide. (n=5, ns, not significant).

**
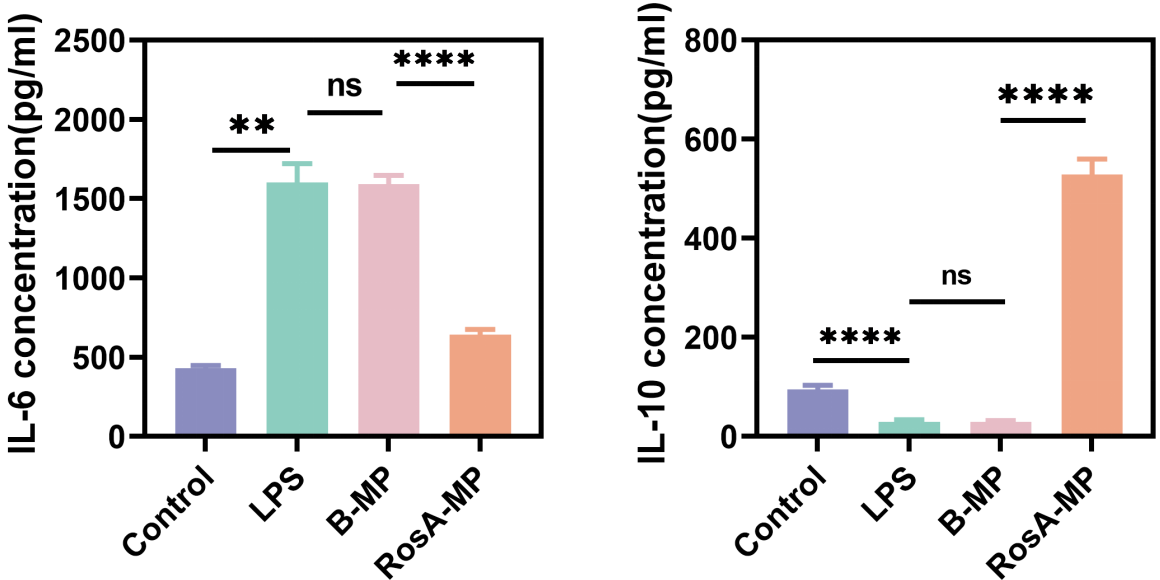
**

**Fig.S10** Concentrations of IL-6 and IL-10 in the supernatant of Raw 264.7 cells. (n=5, ns, not significant; ^**^p < 0.01; ^****^p < 0.001).


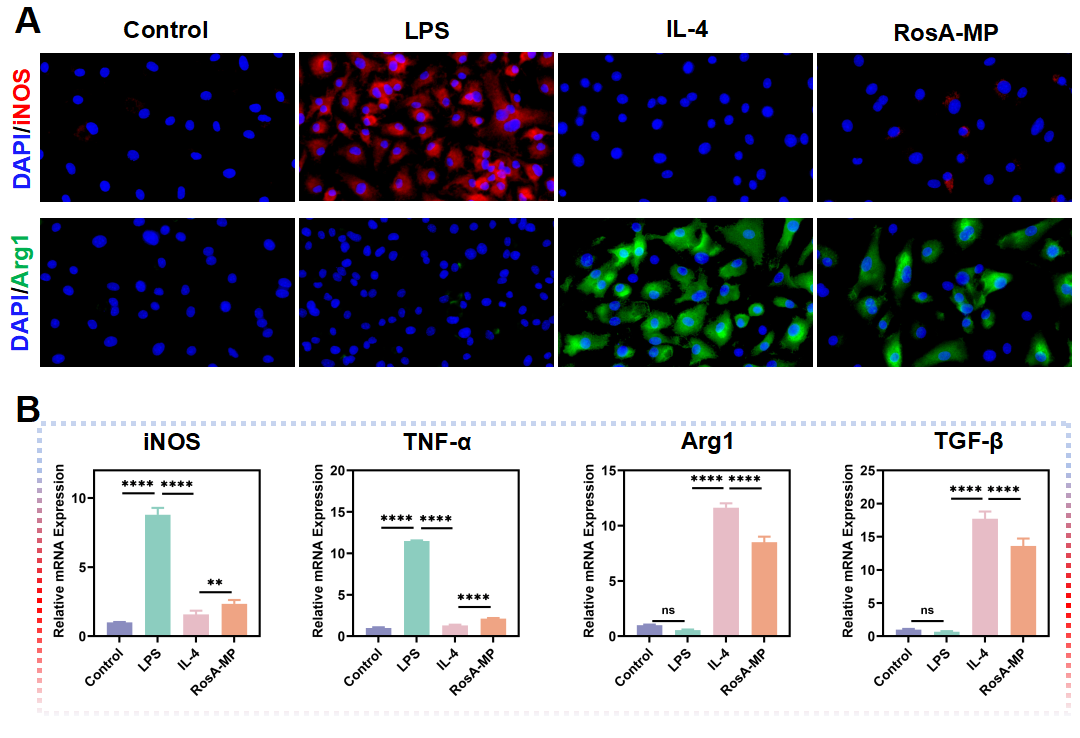


**Fig.S11** The RosA-MP induces M2 polarization in primary mouse BMDMs. (A) Immunofluorescence analysis showing enhanced Arg1 expression in BMDMs following treatment with the RosA-MP and IL-4 compared to both untreated control (M_0_) and LPS (M_1_)-treated groups. (B) Quantitative PCR analysis of M_2_-associated markers (Arg1, TGF-β) and M_1_-associated markers (iNOS, TNF-α). (n=5, ns, not significant; ^**^p < 0.01; ^****^p < 0.001).


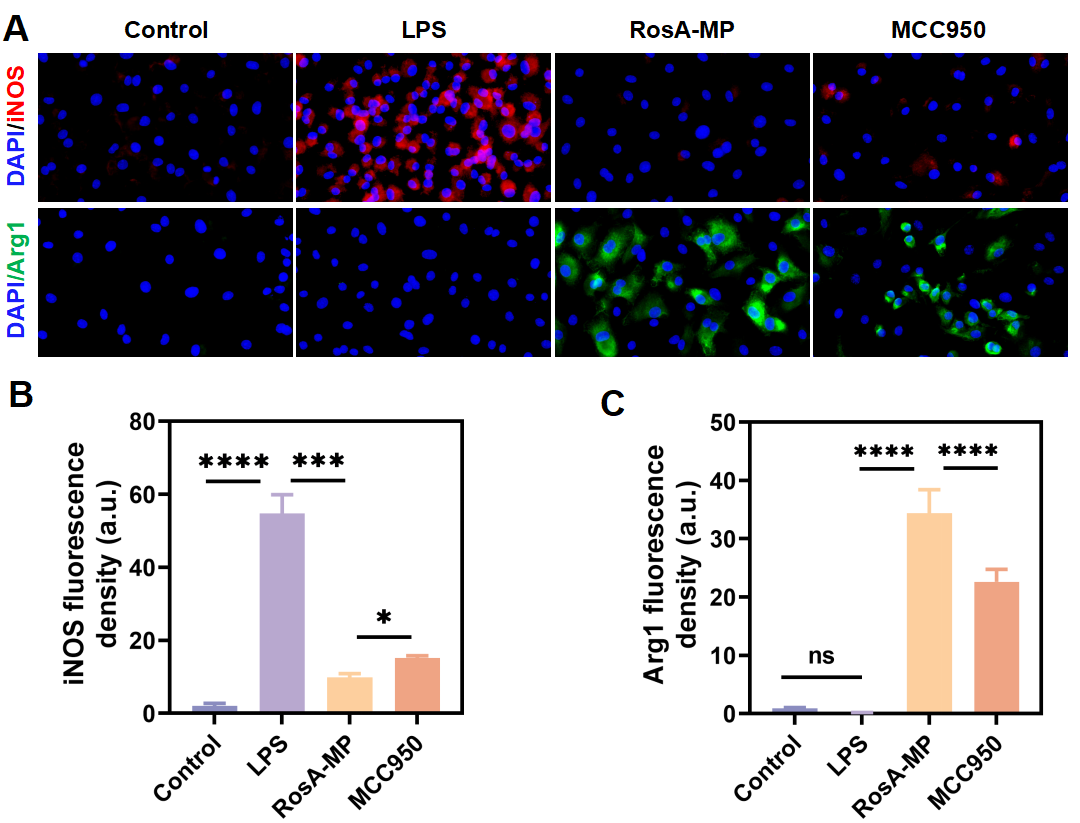


**Fig.S12** BMDMs were treated with the specific NLRP3 inhibitor MCC950. (A) Immunofluorescence analysis reveals that MCC950 treatment substantially increases the expression of the M_2_ marker Arg1, while decreasing the M_1_ marker iNOS. (B, C) (C-E) Corresponding statistical analysis of the fluorescence intensities of iNOS and Arg1. (n=5, ns, not significant; ^*^p < 0.05; ^****^p < 0.001).


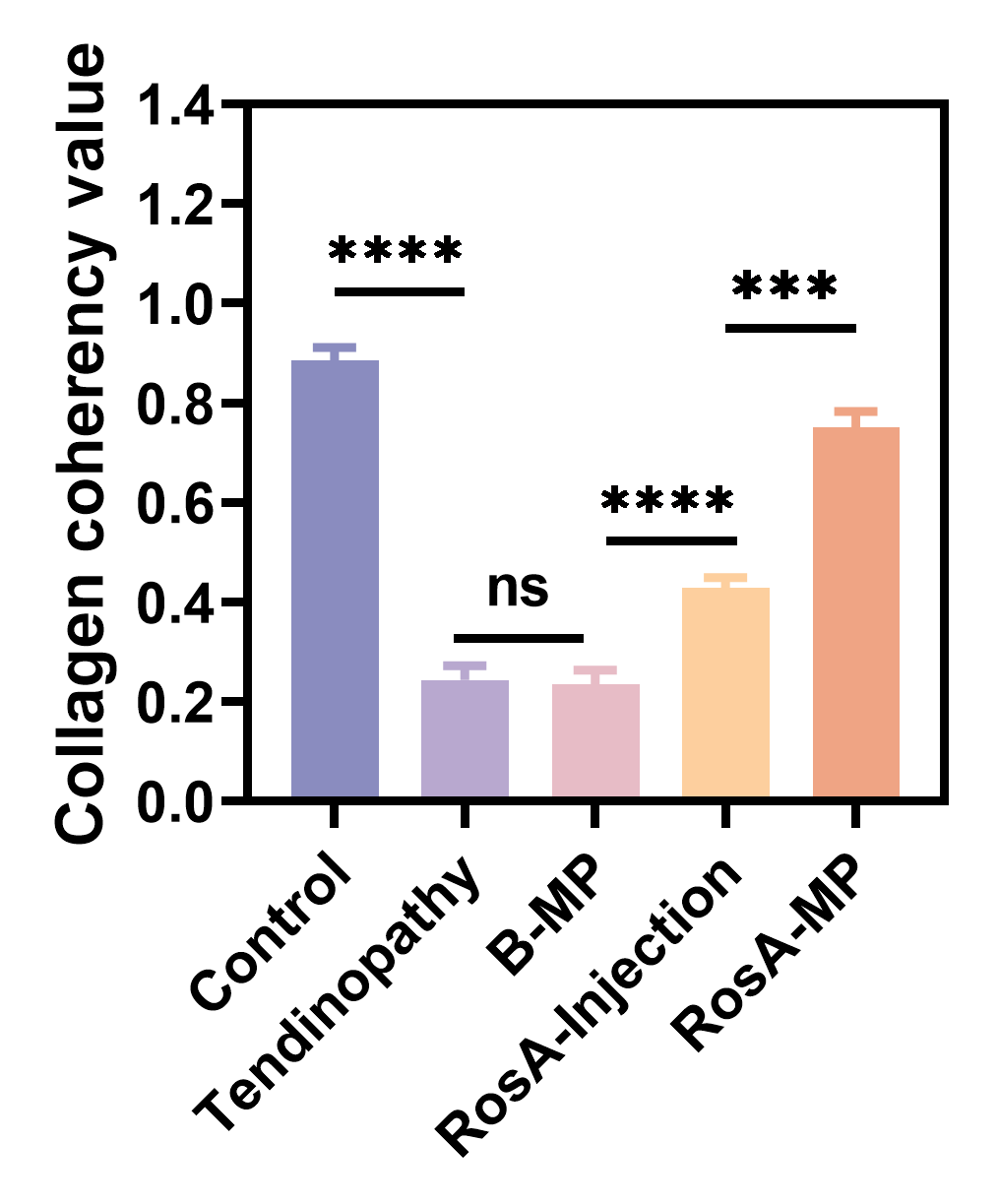


**Fig.S13** Masson trichrome staining of tendon sections was analyzed using ImageJ to assess collagen alignment (coherency value). (n=5, ns, not significant; ^***^p < 0.005; ^****^p < 0.001).


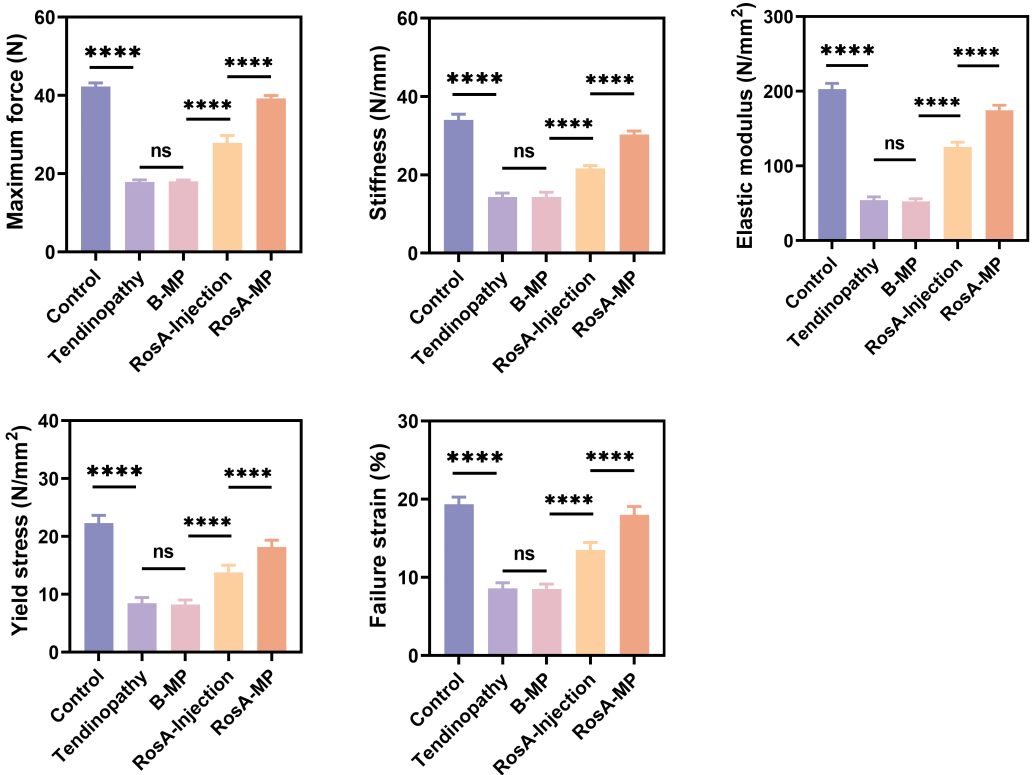


**Fig.S14** Biomechanical properties of tendons in different experimental groups. (n=5, ns, not significant; ^****^p < 0.001).


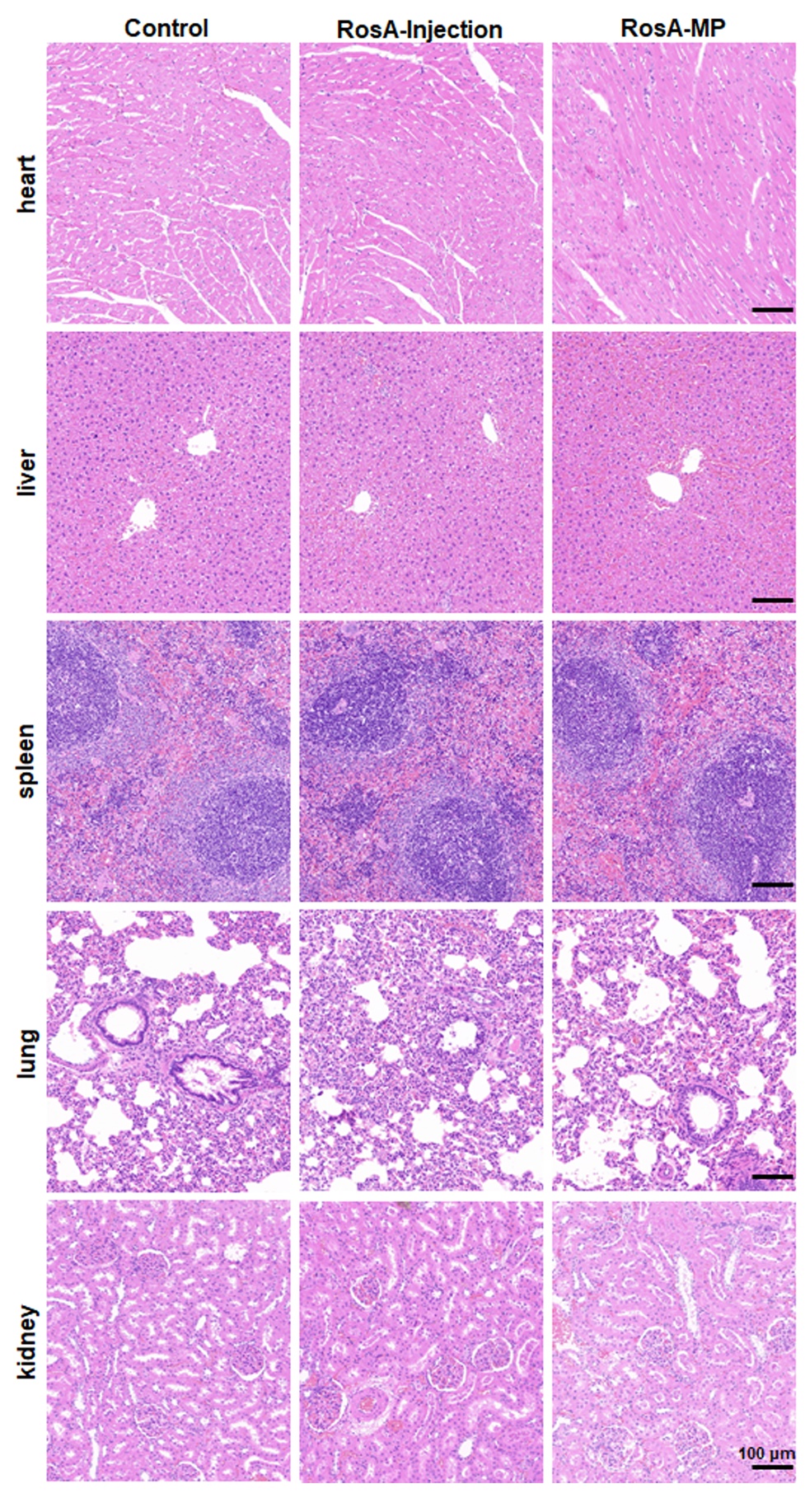
**Fig.S15** H&E staining results of major organs after 4 weeks’ treatment of the RosA-MP.
